# Directly converted astrocytes retain the ageing features of the donor fibroblasts and elucidate the astrocytic contribution to human CNS health and disease

**DOI:** 10.1111/acel.13281

**Published:** 2020-12-13

**Authors:** Noemi Gatto, Cleide Dos Santos Souza, Allan C. Shaw, Simon M. Bell, Monika A. Myszczynska, Samantha Powers, Kathrin Meyer, Lydia M. Castelli, Evangelia Karyka, Heather Mortiboys, Mimoun Azzouz, Guillaume M. Hautbergue, Nóra M. Márkus, Pamela J. Shaw, Laura Ferraiuolo

**Affiliations:** ^1^ Sheffield Institute for Translational Neuroscience (SITraN) University of Sheffield Sheffield UK; ^2^ The Research institute Nationwide Children’s Hospital Columbus OH USA

**Keywords:** ageing, astrocytes, direct reprogramming, in vitro model, neurodegeneration, neuroinflammation, neuron‐astrocyte crosstalk, nuclear abnormalities, nucleocytoplasmic transport, oxidative stress

## Abstract

Astrocytes are highly specialised cells, responsible for CNS homeostasis and neuronal activity. Lack of human in vitro systems able to recapitulate the functional changes affecting astrocytes during ageing represents a major limitation to studying mechanisms and potential therapies aiming to preserve neuronal health. Here, we show that induced astrocytes from fibroblasts donors in their childhood or adulthood display age‐related transcriptional differences and functionally diverge in a spectrum of age‐associated features, such as altered nuclear compartmentalisation, nucleocytoplasmic shuttling properties, oxidative stress response and DNA damage response. Remarkably, we also show an age‐related differential response of induced neural progenitor cells derived astrocytes (iNPC‐As) in their ability to support neurons in co‐culture upon pro‐inflammatory stimuli. These results show that iNPC‐As are a renewable, readily available resource of human glia that retain the age‐related features of the donor fibroblasts, making them a unique and valuable model to interrogate human astrocyte function over time in human CNS health and disease.

## Graphical abstract

iNPC‐As from fibroblasts isolated from donors of different ages retain a spectrum of age‐associated features, such as nuclear morphology and organization, as well as specific nucleo‐cytoplasm shuttling characteristics and oxidative stress response. Moreover, old donor‐derived iNPCs‐As are less supportive to neurons after pro‐inflammatory insult in human co‐culture.

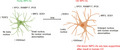

## 1 INTRODUCTION

Ageing is considered the primary risk factor for many devastating pathologies, including neurodegenerative diseases, which primarily affect neurons. There is robust evidence, however, for non‐cell autonomous mechanisms in which neurodegeneration is influenced or even driven by glial cells (Chai & Kohyama, 2019; Domenico et al., 2019; Lee et al., 2016; Lobsiger & Cleveland, 2007; Meyer et al., 2014).

Astrocytes, the most abundant non‐neuronal cell population in the central nervous system (CNS), are known to guide brain development (Verkhratsky & Nedergaard, 2016) and to play a crucial role in CNS homeostasis and repair (Parpura et al., 2012). Moreover, studies interrogating the gene expression profile of rodent and humans neurons, astrocytes and microglia have found that astrocytes, more than neurons, dramatically change their gene expression pattern with age (Soreq et al., 2017). This indicates that they might be significant drivers of the ageing process and preservation of their physiological functions over time will clearly support neuronal health.

Changes in glial functions, such as reduced redox homeostasis and increased pro‐inflammatory responses, are hallmarks of the ageing brain (Bellaver et al., 2017; Lynch et al., 2010; Matias et al., 2019). Consistently, in vitro studies comparing primary astrocytes from young and old rodents have shown changes in the expression of the nuclear factor erythroid‐derived 2‐like 2 (Nrf2) (Duan et al., 2009; Lewis et al., 2015) and nuclear factor kappa B (NFκB) (Osorio et al., 2016; Tilstra et al., 2011), master regulators of the antioxidant and inflammatory response respectively. A decrease in the effectiveness of the antioxidant response in the ageing brain leads to accumulation of oxidised nucleic acids, proteins and lipids (Gemma et al., 2007), while increased NFκB activity exacerbates the production of pro‐inflammatory cytokines (Lynch, 2010; Rea et al., 2018). Both processes are known to participate in neurodegeneration and are therefore considered appealing therapeutic targets.

The lack of human in vitro models that recapitulate the functional changes affecting astrocytes throughout ageing represents a major limitation for studying relevant mechanisms and potential therapies aiming to preserve brain health, as well as targeting age‐related neurodegenerative disorders. Studies using astrocytes isolated from *postmortem* (PM) human samples (Blasko et al., 2000; Re et al., 2014) have shed light on important disease mechanisms, but the availability of these cells is limited and their function might be altered by factors related to *post*‐*mortem* collection, hence the need for widely available, reproducible human in vitro models that retain age‐related biochemical alterations.

So far, somatic cell reprogramming is the most commonly used methodology (Takahashi et al., 2007) to address the challenges of modelling human neurodegenerative diseases. The induction of pluripotency factors in adult fibroblasts, however, reverts cellular age to an embryonic status (Lapasset et al., 2011; Patterson et al., 2012), which is retained even after conversion into neurons, erasing ageing‐associated signatures (Miller et al., 2013). To circumvent this limitation, recent studies have shown that neurons directly reprogrammed from fibroblasts without the use of pluripotency factors retain ageing signatures compared to induced pluripotent stem cells (iPSC)‐derived neurons (Huh et al., 2016; Mertens et al., 2015; Tang et al., 2017; Victor et al., 2018). Direct conversion preserves ageing features in neurons; however, no study has yet achieved this goal in astrocytes. Most available protocols for derivation of human astrocytes utilise iPSCs are time‐consuming and have low conversion efficiency (Almad & Maragakis, 2018). Although recent methodologies have made human astrocyte production from iPSCs faster (Canals et al., 2018; Tchieu et al., 2019), the field is still lacking an in vitro human astrocyte system able to retain the ageing characteristics of the donor.

In 2014, we described the first human‐derived astrocytes differentiated from tripotent‐induced neural progenitor cells (iNPCs) directly converted from adult fibroblasts (Meyer et al., 2014). This protocol is fast and highly efficient and does not involve clonal expansion, thus greatly reducing the variability associated with iPSCs (Mertens et al., 2018; Ortmann & Vallier, 2017). Induced NPCs can be expanded and stored for several passages and are an immediate source of neurons (Webster et al., 2016), oligodendrocytes (Ferraiuolo et al., 2016) and astrocytes. Induced‐NPC derived astrocytes (iNPC‐As) can be obtained from iNPCs in only 7 days and have been utilised to study childhood (Boczonadi et al., 2018) and adult‐onset neurodegenerative disorders, including amyotrophic lateral sclerosis (ALS) (Hautbergue et al., 2017; Meyer et al., 2014; Varcianna et al., 2019).

The ability of this protocol to retain ageing features at transcriptional and functional level had not been interrogated before.

In the present study, we assess the ability of this direct conversion methodology to retain the ageing features of the donor's fibroblasts. Comparing the gene expression profiles of iNPC‐As derived from donors in childhood or adulthood with transcriptomic data from adult *post*‐*mortem* (PM) and foetal astrocytes revealed that iNPC‐As reproduce transcriptional age‐related features. We then showed how iNPC‐As from the two different age groups diverge in relation to a spectrum of age‐associated features, such as accumulation of DNA damage, altered nuclear compartmentalisation, oxidative stress and nucleocytoplasmic shuttling defects. Furthermore, we showed an age‐related differential response of iNPC‐As in their ability to support neurons in co‐culture upon stimulation with the inflammatory cytokine interleukin‐1 beta (IL‐1β).

In conclusion, our results show that age‐related features are retained in iNPC‐As reprogrammed from donor fibroblasts, thus making this model a reliable and accessible tool to interrogate human astrocyte function over the life course in health and disease.

## 2 RESULTS

### 2.1 Efficient differentiation of iNPC‐As from young and old donor fibroblasts

**Figure 1:**
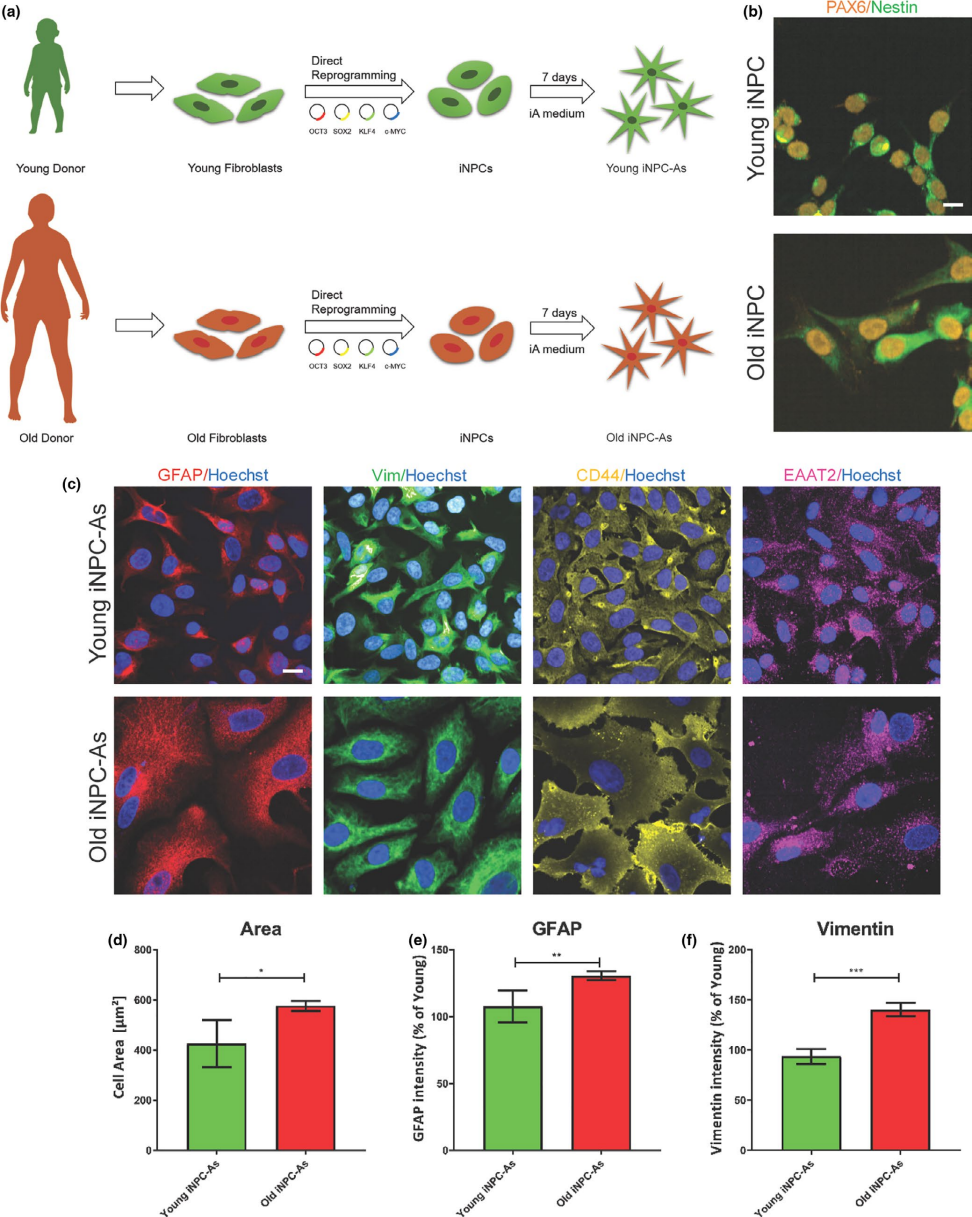
Characterisation of iNPC‐As from old versus young donors. (a) Schematic illustration of the differentiation protocol, from young and old donor fibroblasts to induced neuronal progenitor cell derived astrocytes (iNPC‐As). (b) Representative images of PAX6/Nestin staining in young and old donor‐derived iNPCs. Scale bar (10 µm). See also Figure S1 for markers quantification. (c) Representative images of astrocytic markers (GFAP, vimentin (VIM), CD44 and EAAT2) in young and old donor‐derived iNPC‐As, Scale bar (10 µm). See also Figure S1 for markers quantification. (d) Quantitative image analysis of cell area (µm^2^) in young and old donor‐derived iNPC‐As was assessed using Columbus Software. Unpaired *t* test, *p < 0.05 (*n* = 3/group). (e) GFAP and vimentin (f) intensity quantification in young and old donor‐derived iNPC‐As was assessed using Columbus Software. Unpaired t test, **p < 0.01, ***p < 0.001 (*n* = 3/group)

To determine whether astrocytes derived from iNPCs directly converted from fibroblasts (Meyer et al., 2014) retain the ageing features of the donor, we set out to directly reprogramme fibroblasts from individuals belonging to two distinct age groups. Fibroblast samples from three donors ranging in age from 42 to 56 years and three donors ranging in age from 5 months to 3 years (Table S1) were directly reprogrammed to iNPCs (Figure 1a), which stained positive for the neural progenitor markers Pax6 and Nestin (Figure 1b). Passage matched old and young donor‐derived iNPCs were then differentiated into iNPC‐As in 7 days as previously described (Meyer et al., 2014). iNPC‐As from both young and old donors expressed typical astrocytic markers, including glial fibrillary acidic protein (GFAP), vimentin (VIM), CD44 and the glutamate transporter EAAT2, a marker of mature astrocytes (Figure 1c). This protocol is highly efficient, yielding >98% iNPCs positive for Pax6 and Nestin (Figure S1A,B) and >98% iNPC‐As positive for GFAP, VIM, CD44 and EAAT2 (Figure S1C‐F). Interestingly, iNPC‐As derived from different donors are morphologically heterogeneous one from the other, but display consistent individual morphological characteristics, which are retained throughout different differentiation rounds starting from iNPCs. As noticeable in Figure 1c, astrocytes derived from old donors consistently display a larger cell morphology (Figure 1d), suggesting that older iNPC‐As might be hypertrophic and mildly reactive at baseline, as indicated by the increased expression of GFAP and vimentin (Figure 1e,f). This phenotype has been previously reported in physiologically ageing brains (Sofroniew & Vinters, 2010).

### 2.2 Transcription profile discriminates iNPC‐As from young and old donors and segregates them according to age‐related gene expression signatures

**Figure 2:**
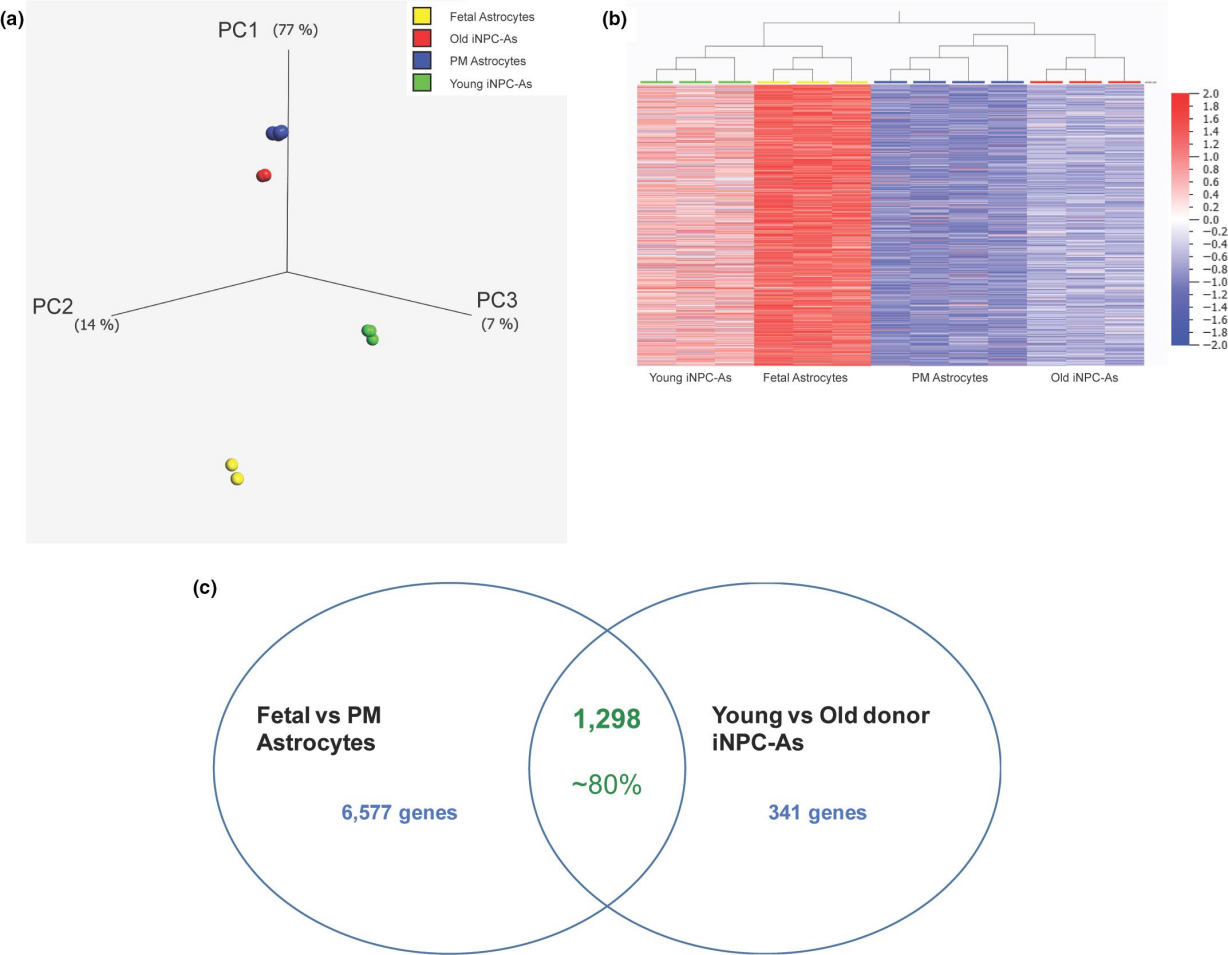
Old donor iNPC‐As and postmortem (PM) astrocytes have distinctly different transcriptomes to foetal astrocytes and young donor iNPC‐As. (a) Principal component analysis (PCA) plot of foetal astrocytes, PM astrocytes and old and young donor‐derived iNPC‐As. Multigroup comparison, *p* ≤ 1 × 10e‐4. (b) Hierarchical cluster heat‐map of foetal astrocytes, PM astrocytes and old and young donor iNPC‐As. Multigroup comparison, *p* ≤ 1 × 10e‐4. (c) Venn diagram representing two‐group comparison analysis (*p*‐value ≤.05; fold change >1.5) between PM and foetal astrocytes as well as iNPC‐As from young and old donors. PM versus foetal astrocytes comparison reported 7875 differentially expressed transcripts, while young and old donors iNPC‐As reported 1639. One thousand two hundred and ninety eight differentially expressed transcripts were common to both comparisons. Approximately 80% of the transcriptional differences between young and old iNPC‐As matched the corresponding comparison between PM and foetal primary human astrocytes. See also Figure S2 and Table S3

To interrogate the transcriptional features of fibroblast‐derived iNPC‐As in relation to their ageing phenotype, we compared transcriptomic data from iNPC‐As obtained from old and young donors to *bona fide* human astrocytes laser captured from *postmortem* (PM) brains (Simpson et al., 2011; Waller et al., 2012) and foetal primary astrocytes (Table S2). After signal normalisation and harmonisation of the four datasets, gene expression levels of 18,357 transcripts were imported into the bioinformatics software Qlucore. The data were then visualised using a principal component analysis (PCA) plot (Figure 2a), with application of stringent multigroup comparison statistics (*p*‐value ≤1 × 10e‐4). This analysis identified 16,284 differentially expressed transcripts that determined sample grouping on the PCA plot (Figure 2a). Using the same parameters, we also visualised the sample groups based on transcript levels in a hierarchical cluster heat map (Figure 2b). Both data representation plots showed that iNPC‐As from the two different age groups have distinct transcription profiles, which group closely with their *bona fide* astrocyte counterpart. In fact, individuals in their 5th‐6th decade of life (old donor) group closely with astrocytes taken at PM from older individuals, while iNPC‐As from individuals in their 1st decade of life (young donor), although clearly separate from foetal astrocytes, group more closely to these samples. In order to determine whether the transcripts described age‐related features, we downloaded a human dataset of 307 genes commonly altered during ageing, from the Human Genomic Resources (HAGR), GenAge database (https://genomics.senescence.info/genes/index.html). From this list of 307 genes, 285 genes were present in the list of 16,284 transcripts that determined the sample subgrouping. Consistently, the PCA plot (Figure S2A) and the hierarchical clustering (Figure S2B) of the four groups in relation to these 285 age‐related transcripts confirmed the separation between PM and foetal astrocytes and iNPC‐As in the two age groups (Figure S2). We also performed two‐group comparison analysis (*p*‐value ≤0.05, fold change >1.5), between *bona fide* astrocytes from different age groups, PM and foetal astrocytes, as well as iNPC‐As from young and old donors. The first comparison (PM vs. foetal astrocytes) reported 7875 differentially expressed transcripts, with only eight transcripts overexpressed in PM astrocytes compared to foetal. The second comparison (young vs. old iNPC‐As) reported 1639 with only five transcripts overexpressed in old iNPC‐As in comparison with the young. We then proceeded to interrogate how many of these differentially expressed transcripts were common to both comparisons, and almost 80% of the transcriptional differences between young and old iNPC‐As, that is, 1298 transcripts, matched the corresponding comparison between PM and foetal primary human astrocytes (Figure 2c) with only two transcripts overexpressed in the old astrocytes compared to young. Pathway analysis of these 1298 transcripts (Table S3) via Panther (http://www.pantherdb.org/) identified several physiological and pathological processes known to be associated with ageing (Table 1).

**Table 1:** Table illustrating the pathways identified by the 1298 transcripts intersecting the comparisons between PM versus foetal astrocytes as well as iNPC‐As from young versus old donors.

Pathway name	% transcripts
Integrin signalling pathway	4.50
Wnt signalling pathway	4.20
Huntington disease	3.40
Inflammation mediated by chemokine and cytokine signalling pathway	3.40
EGF receptor signalling pathway	3.20
DNA damage signalling pathway	2.20
Alzheimer disease‐amyloid secretase and presenilin pathway	2
Cytoskeletal regulation by Rho GTPase	1.90
Parkinson disease	1.90
p53 pathway	1.80
Ubiquitin proteasome pathway	1.80
TGF‐beta signalling pathway	1.60
Oxidative stress response	1.60
Heterotrimeric G protein signalling pathway‐Gi alpha and Gs alpha mediated pathway	1.50
Axon guidance mediated by semaphorins	1.40
PI3 kinase pathway	1.30
VEGF signalling pathway	1.30
Glutamate receptor pathway	1
Insulin/IGF pathway mitogen‐activated protein kinase kinase/MAP kinase cascade	0.90
Protein biosynthesis	0.70
p38 MAPK pathway	0.60

This skew towards transcript downregulation was absent when comparing astrocyte groups of the same age. Only 244 transcripts were dysregulated when comparing PM versus old iNPC‐As (142 up and 102 downregulated), while 1138 transcripts were dysregulated when comparing foetal versus young iNPC‐As, with 972 upregulated and 166 downregulated transcripts in foetal astrocytes. Although this is a preliminary observation, this trend might indicate a progressive suppression of transcription throughout development and ageing (Stegeman & Weake, 2017).

### 2.3 iNPC‐As from older donors recapitulate nuclear ageing features

**Figure 3:**
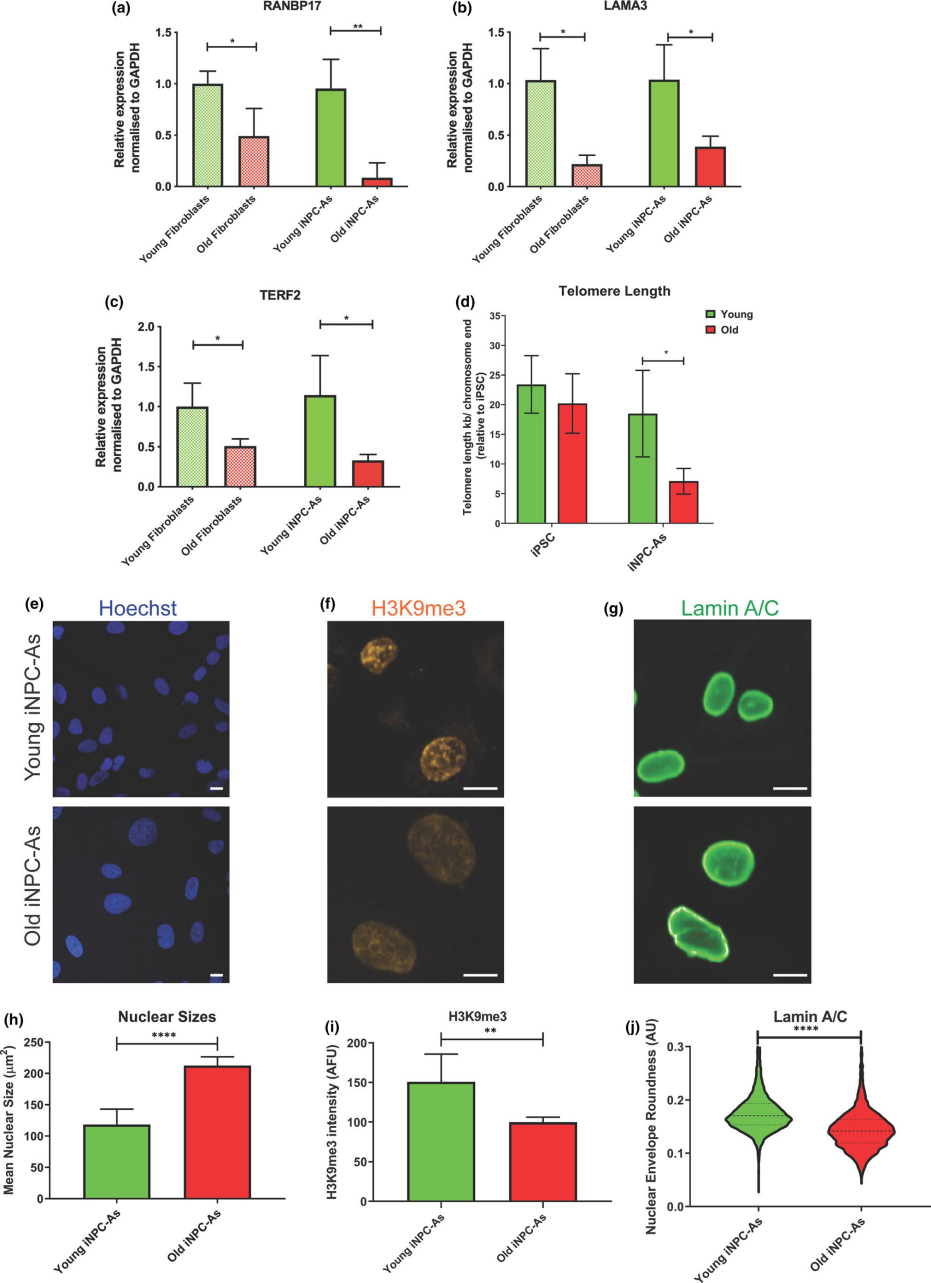
Young and old iNPC‐As retain the ageing phenotype of their fibroblasts of origin. (a) Quantification of *RANBP17*, *LAMA3A* (b) and *TERF2* (c) mRNA levels in young and old donor fibroblasts and derived iNPC‐As Three old and three young iNPC‐As lines were assessed (*n* = 3 biological replicates/group and each line was assessed three times). Error bars = SD; Unpaired *t* test. (d) Telomere length of iPSC cells and iNPC‐derived astrocytes from young and old donors using the Absolute Human Telomere Length quantification qPCR assay Kit. Three old and three young iNPC‐As lines were assessed (*n* = 3 biological replicates/group). Error bars = SD Unpaired *t* test. (e) Representative images of Hoechst, H3K9me3 (f), Lamin A/C (g) staining in young and old donor‐derived iNPC‐As. Scale bar (10 µm). (h) Quantitative image analysis of nuclear sizes(µm^2^), H3K9me3 intensity (i) in young versus old donor‐derived iNPC‐As. Nuclear size was assessed using Columbus Software. Error bars = SD Unpaired *t* test, **p < 0.01, ****p < 0.0001. Mean values per well from three old and three young iNPC‐As lines were assessed (*n* = 3 biological replicates/group); each experiment included three technical repeats and in each well between 500 and 700 cells were assessed. (j) Lamin A/C nuclear envelope roundness. Values from a total of about 3600 cells assessed in each age group, that is, three old and three young iNPC‐As lines (about 1200 cells per line) are plotted to assess the distribution of cell roundness phenotypes. Unpaired *t* test

In order to determine whether the expression of specific age‐associated transcripts was preserved between iNPC‐As and their fibroblasts of origin, we interrogated the expression of three transcripts that have been previously identified as consistently decreasing with ageing in various tissues (Godin et al., 2016; Martínez et al., 2014; Mertens et al., 2015). RAN binding protein 17 (*RANBP17*) and laminin subunit alpha 3A (*LAMA3A*) were identified by Mertens *et al* when comparing the transcriptomes of human fibroblasts and brain samples from a broad range of aged donors. Their change in expression was shown to be maintained between fibroblasts and derived directly reprogrammed neurons (iNeurons), but not iPSC‐derived neurons. RANBP17 is a nuclear pore‐associated transport receptor, a member of the importin‐β family, involved in the transport of nuclear localisation signal (NLS)‐containing cargo proteins through the nuclear pore complex (NuPC) (Koch et al., 2000; Lee et al., 2010), while *LAMA3*, also reported to decrease with age in other in vivo studies (Godin et al., 2016), encodes an extracellular matrix protein. In addition, we also assessed the expression of telomeric repeat‐binding factor 2 (TERF2), a component of shelterin that together with TERF1 is responsible for telomerase maintenance (Martínez et al., 2014), and telomere length (Smogorzewska et al., 2000). It is well known that telomerase activity is crucial in determining telomere length in ageing cells and that telomere length can be considered as a biomarker of chronological ageing (Fasching, 2018; Rizvi et al., 2015). In agreement with previous reports, our results show that *TERF2*, *RANBP17* and *LAMA3* mRNA levels decrease significantly with age in fibroblasts and we show here that iNPC‐As retain this decline in expression levels after reprogramming (Figure 3a‐c). Consistent with the age‐related decline in *TERF2*, qPCR data of genomic DNA show a significant difference between telomere length in young and old iNPC‐As (Figure 3d). As a control, we also assessed telomere length in iPSCs derived from a different set of fibroblast donors 1 month and 55 years of age, and, as expected, we found no difference in their telomere length regardless of donor's age.

In addition to assessment of transcriptional features, we interrogated cellular parameters that have previously been reported to change with age, including nuclear size and shape (Haithcock et al., 2005; Pienta et al., 1992), as well as histone methylation (Miller et al., 2013).

It is well documented how nuclear structure is affected during ageing (Haithcock et al., 2005; Scaffidi et al., 2005). Heterochromatin organisation and nuclear shape were assessed via immunostaining using the heterochromatin‐associated trimethylated ‘Lys‐9’ on histone H3 marker (H3K9me3) and the nuclear lamina marker lamin A/C. H3K9me3 is the hallmark of constitutive heterochromatin (Maleszewska et al., 2016), while Lamin A/C coded by the gene *LMNA*, is part of the lamin protein family, which form components of the nuclear lamina (Liu & Zhou, 2008).

Consistent with previous reports describing an overall decrease in H3K9me3 in cells and tissues (Miller et al., 2013) iNPC‐As from older donors displayed a significant global reduction of H3K9me3 staining (Figure 3f,i). Immunostaining of lamin A/C also confirmed that iNPC‐As from older donors displayed significant nuclear morphology alterations, with increased nuclear folding and blebbing. Lamin staining is clearly localised in the nuclear membrane and defines nuclear shape in young iNPC‐As, while it displays fragmented and less spherical staining in iNPC‐As from older donors (Figure 3g,j). In addition, we report a significant increase in nuclear size assessed by Hoechst staining in old iNPC‐As (Figure 3e,h). Similar findings have been reported in in vitro and in vivo fibroblasts (Mukherjee & Weinstein, 1986; Pienta et al., 1992).

Taken together, these results demonstrate that iNPC‐As reprogrammed from donors of different age groups retain some of the ageing features of their parental fibroblasts and exhibit well‐described ageing morphological characteristics.

### 2.4 iNPC‐As reveal age‐related nuclear permeability and nucleocytoplasmic transport defects

In order to examine further the nuclear envelope abnormalities detected via lamin A/C staining, we set out to interrogate the overall integrity of another nuclear component, the nuclear pore complex (NucPC). Seminal work (D’Angelo et al., 2009) has previously revealed that the NucPC is highly affected by age in post‐mitotic cells, leading to nuclear ‘leakiness’. This phenomenon has also been recently highlighted as critically important in age‐related diseases, such as amyotrophic lateral sclerosis (ALS) and Alzheimer's disease (AD) (Eftekharzadeh et al., 2018; Kim & Taylor, 2017).

**Figure 4:**
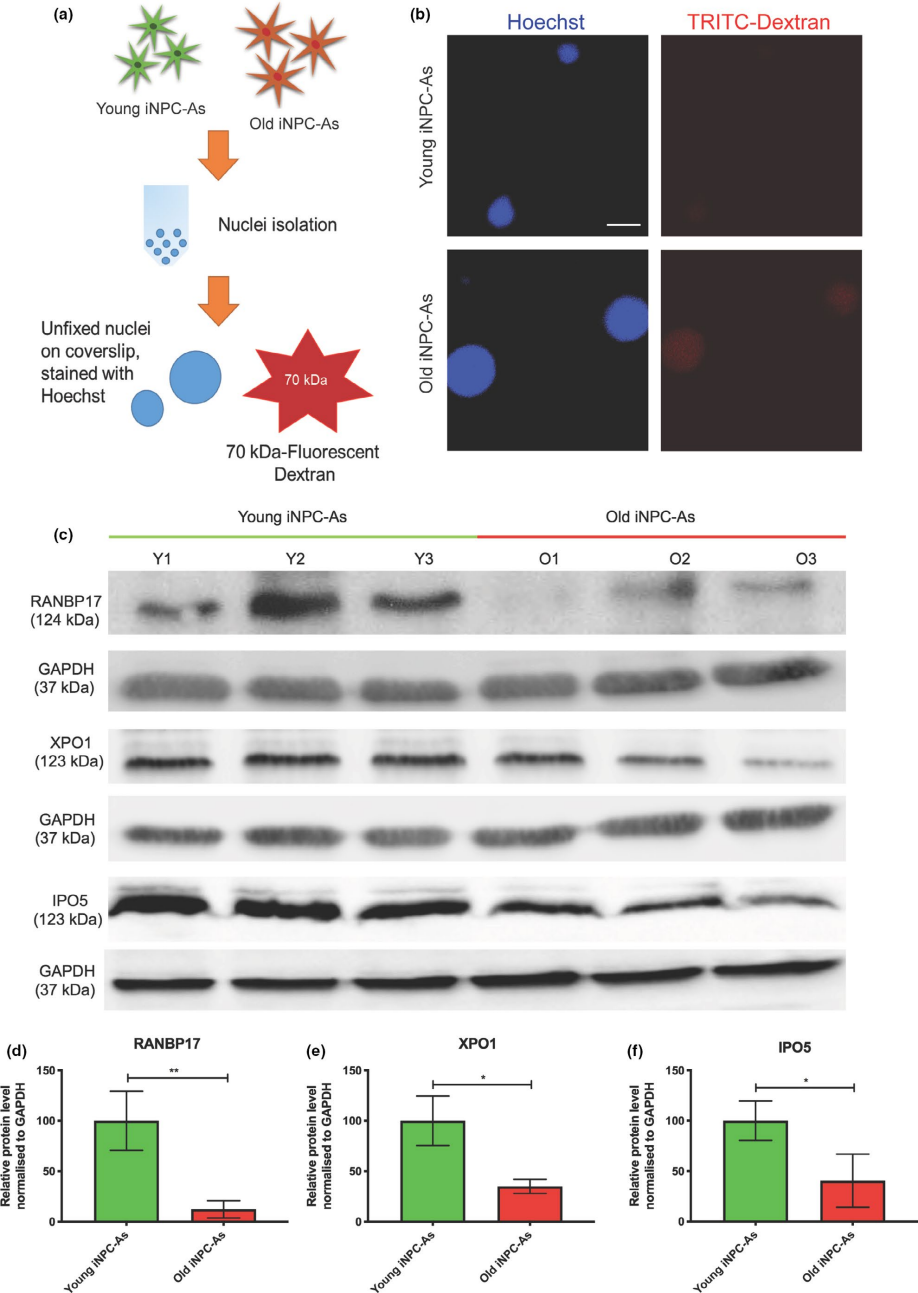
Old and young iNPC‐As show differential nuclear permeability and nucleocytoplasmic shuttling/export protein amounts. (a) Schematic illustration of the nuclear isolation protocol for dextran application. (b) Representative images of TRITC‐labelled dextran influx in young versus old donor‐derived iNPC‐As. (c) Representative images of western blot analysis of RANBP17, IPO5 and XPO1. Protein from young and old donor‐derived iNPC‐As was separated by sodium dodecyl sulphate‐polyacrylamide gel electrophoresis followed by immunoblotting. (d) Densitometric quantification of RANBP17 normalised to GAPDH. Unpaired *t* test, **p < 0.01 (*n* = 3). (e) Densitometric quantification of XPO1 normalised to GAPDH. Unpaired *t* test, *p < 0.05. (*n* = 3). (f) Densitometric quantification of IPO5 normalised to GAPDH. Unpaired *t* test, *p < 0.05 (*n* = 3). Each old and young iNPC‐As sample (*n* = 3/group) was assessed in three independent experimental repeats and results are expressed as the mean of the three repeats/sample. Error bar ± SD.

In intact nuclei, molecules of up to 40–50 kDa can pass through the NucPC via passive diffusion, while molecules larger than 60 kDa are excluded from intact nuclei (Lénárt & Ellenberg, 2006). Thus, we analysed the overall nuclear permeability in young and old donor iNPC‐As, by isolating nuclei from both groups of iNPC‐As and incubating them with a 70 kDa fluorescently labelled dextran (Figure 4a). Nuclei were then stained for Hoescht and imaged using a confocal microscope. We found that influx of the 70 kDa dextran was observed only in the old donor‐derived iNPC‐A nuclei (Figure 4b), and not in young donor‐derived iNPC‐A nuclei. This confirms that direct conversion of fibroblasts into iNPCs and subsequently iNPC‐As retains a key characteristic of ageing that is often associated with neurodegenerative conditions (D’Angelo et al., 2009) and so far has been primarily associated with neuronal ageing.

Further, we assessed the levels of three proteins involved in active nucleocytoplasmic transport, that is, RANBP17, Importin 5 (IPO5) and Exportin 1 (XPO1). IPO5 is a member of the importin‐β family of nuclear transport receptors that mediates transport of proteins carrying a nuclear localisation signal (NLS), RANBP17 serves as nuclear import receptor, while XPO1 mediates the nuclear export of cellular proteins bearing a leucine‐rich nuclear export signal (NES) and of some non‐coding RNA molecules including snRNAs and rRNAs.

Consistent with reports that nucleocytoplasmic transport becomes impaired with age and in neurodegeneration (Ferreira, 2019; Hutten & Dormann, 2019; Kim & Taylor, 2017; Ribezzo et al., 2016), our results show that RANBP17, IPO5 and XPO1 protein levels are significantly decreased in older compared to young donor‐derived iNPC‐As (Figure 4c–f).

### 2.5 Older donor‐derived iNPC‐As display lower antioxidant defences

**Figure 5:**
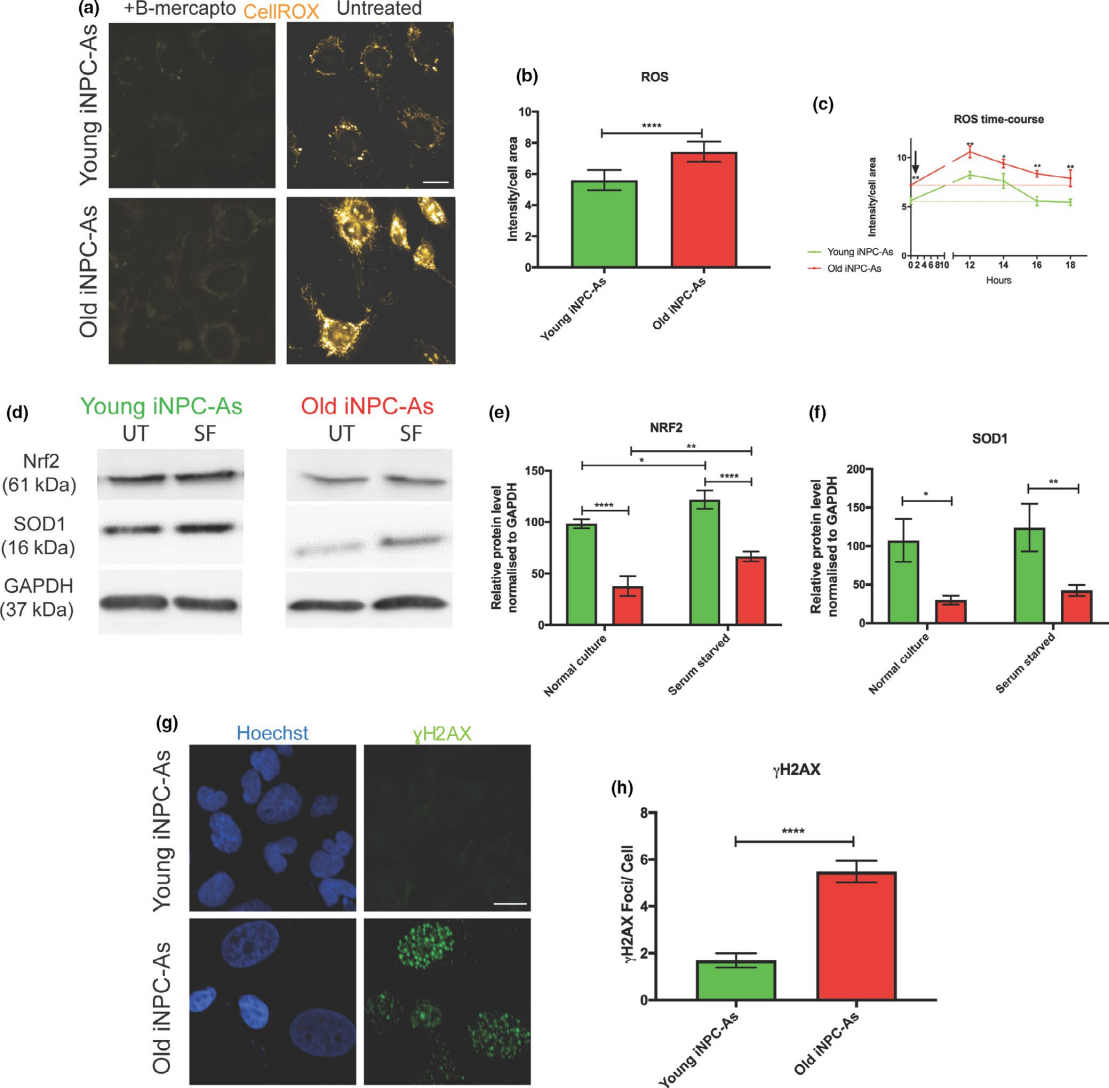
Altered oxidative stress capacities in old donor‐derived astrocytes. (a) Representative immunofluorescence images of ROS in young and old donor‐derived iNPC‐As at baseline or treated with 0.0016% V/V of β‐mercaptoethanol in culture medium as antioxidant negative control. Cellular ROS levels were assessed in iNPC‐As by CellROX^®^ Reagent, Scale bar (10 µm). (b) Quantitative image analysis of ROS detected by CellROX^®^ Reagent in young and old donor‐derived iNPC‐As. Fluorescence intensity was assessed using Columbus Software. Unpaired *t* test, ****p < 0.0001 (*n* = 3). (c) Quantitative imaging analysis of ROS time‐course detected by CellROX^®^ Reagent in young and old donor‐derived iNPC‐As. Fluorescence intensity was assessed using Columbus Software. Unpaired *t* test between young and old iNPC‐As at each timepoint, **p < 0.01, *p < 0.05 (*n* = 3). For the CellROX data, each old and young iNPC‐As sample (*n* = 3/group) was assessed in three independent experimental repeats each containing three technical replicates. Results are expressed as the mean of the three experimental repeats/sample. Error bar ± SD. (d) Representative images of Western blot analysis of NRF2 and SOD1. Cells were incubated in serum‐free media for 24 h and harvested (UT = untreated, SF = serum‐free media). Protein from young and old donor‐derived iNPC‐As was separated by sodium dodecyl sulphate‐polyacrylamide gel electrophoresis followed by immunoblotting. (e) Densitometric quantification of NRF2 and SOD1 (F) protein levels, normalised to GAPDH. Two‐way ANOVA, ****p < 0.0001, *p < 0.05, **p < 0.01. For the WB data, each old and young iNPC‐As sample (*n* = 3/group) was assessed in three independent experimental repeats and results are expressed as the mean of the three repeats/sample. Error bar ± SD. (g) Representative images of yH2AX staining and foci/cell quantification (H) in young and old donor‐derived iNPC‐As, Scale bar (10 µm). Results are expressed as the means of three independent experimental repeats/sample, each containing three technical replicates/experiment. Error bars ± SD. Unpaired *t* test, ****p < 0.0001 (*n* = 3).

A key hallmark of ageing is rising levels of oxidative stress and accumulation of reactive oxygen species (ROS) that leads to nucleic acid, protein and lipid oxidation. Astrocytes are the principal cell type in the CNS responsible for counteracting the increase in oxidative stress through the antioxidant response and noncell autonomous support of neurons (Bell et al., 2011). To test the hypothesis that reprogrammed iNPC‐As from donors of different ages can model this central aspect of astrocyte function, we quantified the endogenous levels of ROS in iNPC‐As from naïve young and old donors, using the CellROX^®^ Oxidative Stress Reagent, a fluorogenic probe designed to measure ROS in live cells. We found that ROS levels were significantly higher in old compared to young donor‐derived iNPC‐As under basal conditions (Figure 5a,b). To test how iNPC‐As respond to stressors that increase oxidative stress over time, we also performed a time‐course experiment. We measured intracellular levels of ROS in young and old donor iNPC‐As at baseline before subjecting the cells to 12 h of serum starvation. After 12 h, we measured the levels of intracellular ROS in both groups of iNPC‐As, and then following a change to full iNPC‐A medium assessed how long it took the cells to return to baseline levels of ROS. Our measurements show that both young and old donor‐derived iNPC‐As display an increase in intracellular ROS after serum starvation and recover after removing the stimulus, going back to baseline levels (Figure 5c). Remarkably, iNPC‐As from younger individuals recovered to their baseline levels after only 4 h, while iNPC‐As from older individuals required a longer time‐period of 6 h (Figure 5c).

To understand what changes may underlie the increased levels of ROS seen in old iNPC‐As at baseline, and the delayed recovery from oxidative stress following serum starvation, we investigated the levels of NRF2, the master regulator of antioxidant defences, and superoxide dismutase 1 (SOD1), a major antioxidant enzyme responsible for the breakdown of superoxide radicals. Both *NRF2* and *SOD1* are present on the list of ageing genes that are downregulated in both PM and old iNPC‐As versus foetal astrocytes and young iNPC‐As in our microarray analysis (Figure 2 and Figure S2B). Consistent with in vivo data (Paladino et al., 2018) and our transcriptomic data, we found that NRF2 and SOD1 baseline levels are significantly lower in old as opposed to young donor‐derived iNPC‐As (Figure 5d–f). The lowered antioxidant defences present in old iNPC‐As may explain the increased levels of baseline ROS species observed (Figure 5a,b). Considering the ability of both young and old donor iNPC‐As to respond to the oxidative stress generated by serum starvation treatment (Figure 5c), we set out to evaluate the protein levels of NRF2 and SOD1 in response to this stressor. As suggested by our time‐course experiments (Figure 5c), we observed a significant increase in NRF2 protein level in iNPC‐As from both age groups, thus showing that both groups can respond to acute oxidative stress insults. However, the total levels of NRF2 and SOD1 were still lower in old compared to young iNPC‐As in the serum‐starved conditions, giving a potential explanation for the decreased and slower response to counteract oxidative stress after the insult (Figure 5d–f).

Oxidative stress is known to affect cell function in many ways through protein, lipid and nucleic acid oxidation. In particular, DNA damage has been recognised as a causal factor in the ageing process and its markers are hallmarks of aged tissues, especially in the CNS, where cell turnover is limited (Lu et al., 2004). The current hypothesis is that DNA damage accumulation with ageing causes loss of key cellular functions leading to degeneration (Ribezzo et al., 2016). In order to repair these lesions, in particular DNA double‐strand breaks (DSBs), the DNA damage response (DDR) starts at the damage site with the phosphorylation of the C‐terminal of the core histone protein H2AX (γH2AX). Consistent with the increase in ROS and concomitant decrease in the antioxidant response, we detected an increase in γH2AX foci in old compared to young iNPC‐As (Figure 5g,h).

### 2.6 Older donor‐derived iNPC‐As are less supportive to MNs upon pro‐inflammatory stress

So far, our results demonstrate that iNPC‐As derived from fibroblasts of donors from different age groups successfully recapitulate important transcriptional and functional age‐related features. To probe the ageing astrocyte phenotype in more depth, we wanted to assess one of the most important functions fulfilled by astrocytes in vivo, that is, their ability to support neurons throughout the ageing process and under stressful conditions. In particular, recent studies have shown that, as astrocytes are exposed to microglia‐secreted pro‐inflammatory cytokines in the ageing brain (Liddelow et al., 2017), they become less supportive to neurons. Therefore, we tested the effect of IL‐1β treatment, as this pro‐inflammatory cytokine is released by microglia in the CNS during ageing (Clarke et al., 2018) and after injury (Pineau & Lacroix, 2007).

To functionally test our cell model, both young and old donor‐derived iNPC‐As were exposed to IL‐1β for 6 h, washed to remove any residual cytokine and were then co‐cultured with human iPSC‐derived motor neurons (Figures S3 and S4) to test their ability to support neurons when challenged with inflammatory stress.

**Figure 6:**
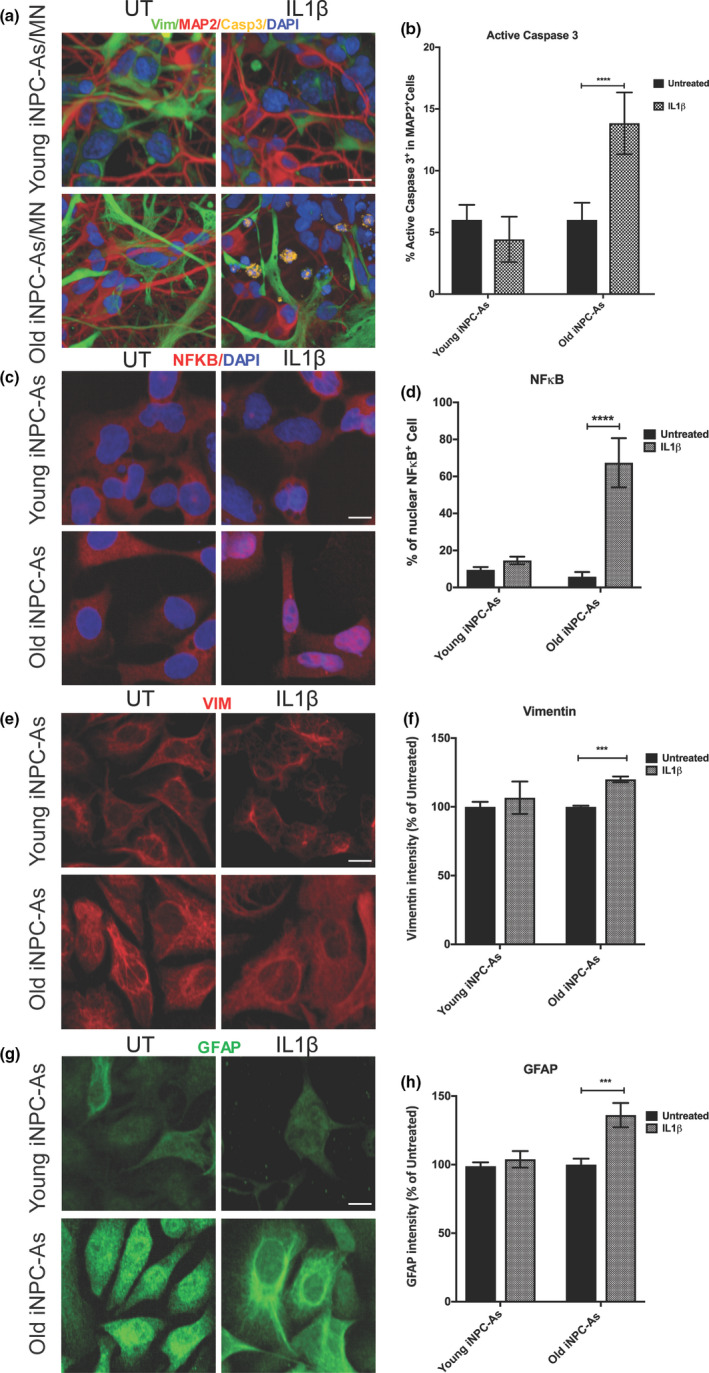
Old donor‐derived iNPC‐As are less supportive to MNs after pro‐inflammatory stress with IL‐1β. (a) Representative images of human MN and young or old donor‐derived astrocytes co‐culture. Scale bar (10 µm). (b) Quantitative imaging analysis of percentage of caspase‐3 in MAP2+ cells. Two‐way ANOVA, ****p < 0.0001. (c) Representative images of NFκB staining and NFκB nuclear activation (d) in untreated and treated with IL‐1β (20 ng/ml) young and old donor‐derived iNPC‐As. Scale bar (10 µm). Two‐way ANOVA, ****p < 0.0001. (e) Representative images and quantitative analysis (f) of vimentin staining in untreated and treated with IL‐1β (20 ng/ml) young and old donor‐derived iNPC‐As. Scale bar (10 µm). Two‐way ANOVA, ****p < 0.001. (g) Representative images and quantitative analysis (H) of GFAP staining in untreated and treated with IL‐1β (20 ng/ml) young and old donor‐derived iNPC‐As. Scale bar (10 µm). Two‐way ANOVA, ***p < 0.001. Results are expressed as the means of three independent experimental repeats/sample (*n* = 3/group), each containing three technical replicates/experiment. Error bars ± SD. In each experiment, at least 150 cells/well were assessed

Caspase‐3, a master regulator of apoptosis, that catalyses the cleavage of many key cellular proteins, was used to assess neuronal health in co‐culture. Immunostaining data showed that both young and old donor‐derived iNPC‐As are equally able to support neuronal survival under basal conditions (Figure 6a,b). Treatment of iNPC‐As with IL‐1β, however, led to a significant 3‐fold increase of caspase‐3 and apoptotic nuclear fragmentation in MAP2^+^ cells cultured with old donor iNPC‐As (Figure 6a,b). In contrast, neurons cultured with young donor iNPC‐As did not show any difference between conditions, that is, iNPC‐As untreated or pretreated with IL‐1β (Figure 6a,b). In order to understand the causes of this differential response, we assessed the effect of IL‐1β treatment on iNPC‐As, with a particular focus on NFκB. NFκB is a transcription factor that regulates multiple aspects of innate and adaptive immune functions and represents a central mediator of inflammatory responses. Our results show that IL‐1β does not affect astrocytes survival (Figure S5); however, it cause a 4‐fold increase in nuclear activation of NFκB compared to young donor iNPC‐As (Figure 6D). Furthermore, old donor iNPC‐As become more reactive, displaying higher levels (intensity/pixel) of GFAP and vimentin compared to their baseline expression level (Figure 6e‐h).

## 3 DISCUSSION

Astrocytes are the largest group of glial cells in the CNS and are responsible for vital homeostatic functions. Several studies have reported that glial cells are potentially more affected by the ageing process than neurons. In particular, astrocytes present dramatic transcriptional (Soreq et al., 2017) and functional changes during ageing (Gemma et al., 2007; Lynch et al., 2010; Rea et al., 2018) that hinder their ability to maintain homeostasis in the CNS and support neurons throughout their life course.

Ageing is a leading risk factor for multiple neurodegenerative diseases, so it is fundamental to recapitulate age‐related characteristics in cells that contribute to and are actively involved in diseases. It is well known that ageing and neurodegeneration share common mechanisms including increased oxidative stress, excitotoxicity and inflammation. Alzheimer's disease (AD), Parkinson's disease (PD) and amyotrophic lateral sclerosis (ALS) are three common adult‐onset neurodegenerative diseases known to involve astrocyte dysfunction (Booth et al., 2017; González‐Reyes et al., 2017; Haidet‐Phillips et al., 2011; Meyer et al., 2014). As a consequence, lack of human in vitro models that mimic the functional features of astrocytes in young and old age is a limitation to studying mechanisms and potential therapies involved not only in maintaining brain health, but also in combatting neurodegenerative diseases and other neurological conditions.

In this study, we successfully derived iNPC‐As from young donors (5 months‐3 years) and compared them to iNPC‐As derived from older donors (42–56 years). Comparative transcriptomic analysis demonstrated that the transcription profile of iNPC‐As from old donors recapitulates transcriptional features of laser‐captured PM astrocytes from adults of a similar age. Although this comparison is affected by the different origin of the cells (ex vivo vs. in vitro) and their neighbouring environment (enriched laser‐captured cell population vs. pure cell population), strikingly, the transcriptional differences between iNPC‐As from young and old donors display ~80% overlap with the differences discriminating between PM and foetal primary human astrocytes. This indicates that iNPC‐As derived from directly converted iNPCs retain astrocyte‐relevant ageing transcriptomic features. In addition, we observed a consistent skew in gene expression upregulation in young astrocytes compared to their older counterpart, suggesting an effect of age on transcription. A similar pattern was also recently reported in a transcriptomic study comparing young and old donor fibroblasts (Sarkar et al., 2020). When we specifically looked at the pathways described by these transcriptional changes, we identified multiple pathways involved in ageing and senescence, that is, p53 signalling, oxidative stress, DNA damage and inflammatory response. Indeed, we confirmed these transcriptional changes at functional level. We show here that direct conversion of fibroblasts to iNPCs and differentiation to iNPC‐As retains key age‐related cellular characteristics. TERF2, an enzyme involved in telomere organisation, decreases significantly with age in fibroblasts and derived iNPC‐As recapitulate this decrease in expression level following reprogramming. Consistently, telomere length is lower in old iNPC‐As. Moreover, our model reproduces the global loss of the heterochromatin marker (H3K9me3) and nuclear organisation abnormalities reported in ageing and neurodegenerative conditions (Miller et al., 2013; Tang et al., 2017). Major Lamin A disruption occurs in Hutchinson‐Gilford progeria syndrome (HGPS), as a consequence of mutations in the gene encoding for this protein, that is, *LMNA*. HGPS is a rare genetic condition that leads to accelerated ageing and brain development abnormalities (Gonzalo et al., 2017), demonstrating the link between nuclear envelope integrity, ageing and neurodegeneration. At a cellular level, HGPS displays a number of characteristics typical of ageing cells including loss of heterochromatin, nuclear lobulation and increased DNA damage (Ghosh & Zhou, 2014). Expression of progerin, a truncated form of LMNA, is used to induce ageing in iPSC models (Miller et al., 2013). Although this approach recapitulates some aspects of cellular ageing, it still carries the substantial limitation of introducing a pathogenic mutation to model a physiological process. In addition, this strategy might create confounding phenotypes when applied in the study of neurodegenerative conditions other than HGPS.

Without inserting any mutation, our data show that iNPC‐As derived from directly reprogrammed iNPCs from older individuals display decreased histone H3 K9 trimethylation, accumulation of DNA damage and mild Lamin A/C disruption with associated loss of nuclear integrity, leading to nuclear ‘leakiness’. This phenotype has been reported in several ageing studies and is associated with loss of integrity of the NuPC (D’Angelo et al., 2009; Lord et al., 2015), thus favouring uncontrolled protein diffusion and loss of the normal compartmentalisation between the nucleus and the cytoplasm.

In addition to this impairment, the present report reveals a significant decrease in three nucleocytoplasmic transporters, RANBP17, IPO5 and XPO1, which play major roles in regulating nuclear import and export. Mertens et al. (2015) have previously reported that RANBP17 expression decreases in ageing cells and directly reprogrammed neurons from old donor fibroblasts, but not in iPSCs. Our data reveal that the age‐related decrease in nucleocytoplasmic transport is bidirectional and is not limited to RANBP17. Nucleocytoplasmic transport has recently emerged as a key dysregulated pathway in neurodegenerative disorders. Several studies have shown how mutations in genes causing amyotrophic lateral sclerosis and frontotemporal dementia (ALS/FTD) lead to aberrant nucleocytoplasmic partitioning of ALS‐causing gene products and that this dysregulation includes both subcellular mislocalisation and cytoplasmic inclusions (Ferreira, 2019). For instance, it has been reported how mutations in TAR DNA‐binding protein 43 (*TDP43*) and Fused in Sarcoma (*FUS*) lead to accumulation of these proteins within the cytoplasm and mislocalisation from the nucleus, thus affecting nucleocytoplasmic transport (Mackenzie et al., 2010). More recently, studies also revealed that the GGGGCC repeat expansion in C9orf72 compromises nucleocytoplasmic transport (Freibaum et al., 2015; Jovičić et al., 2015). Further evidence revealed that this pathway is also impaired in AD (Eftekharzadeh et al., 2018) and Huntington's disease (HD) (Gasset‐Rosa et al., 2017; Grima et al., 2017). Specifically, XPO1 has been identified as an important transporter for many proteins involved in neurodegenerative diseases (Chan et al., 2011; Ederle et al., 2018; Zhong et al., 2017). XPO1 is an essential nuclear export receptor (Stade et al., 1997), which recognises cargoes with the leucine‐rich nuclear export sequence (NES) (Dong et al., 2009). Recently, a study in *C. elegans* showed that ALS‐linked mutant SOD1, when misfolded, exposes a normally buried NES‐like sequence, leading to XPO1‐dependent nuclear export. This has been proposed as a potential protective mechanism against misfolded SOD1 proteotoxicity (Zhong et al., 2017), which, in light of our data, is likely to decrease with age, thus shifting the balance between accumulation of toxic proteins and neuroprotective cellular defences. A similar mechanism has also been reported for CAG repeats expansions associated with polyglutamine (polyQ) diseases, in which exportin 1 regulates the nucleocytoplasmic distribution of expanded polyQ protein (Chan et al., 2011). Decreased expression of XPO1 protein levels in vivo contributes to the accumulation of expanded polyQ protein in the nucleus of symptomatic polyQ transgenic mice. This process drives the nuclear accumulation of the disease protein, and thus, the cell nucleus becomes an important site of pathology in polyQ diseases, where transcription is affected.

Additional stress that can significantly affect transcription, and also translation, is represented by accumulation of ROS that damage lipids, protein and nucleic acids, which are commonly detected as biochemical footprints in aged tissue (Brawek et al., 2010; Lukiw et al., 2012). Oxidative stress is caused by an imbalance in the redox state of the cell, by overproduction of ROS or by impairment of the antioxidant defence system. In particular, the CNS is vulnerable to the effects of ROS due to its high demand for oxygen, and its abundance of substrates highly susceptible to oxidative modification. Neurons are postmitotic and have a very high energy demand, and thus are vulnerable to oxidative insults. Consistently, it has been shown that Nrf2 levels and activity are significantly higher in the longest‐lived rodents compared to short‐lived mice (Lewis et al., 2015). Surprisingly, however, neurons have weak endogenous antioxidant defences, as the main antioxidant pathways such as the Nrf2 pathway are silenced during development within neurons (Bell et al., 2015). Therefore, neurons rely heavily on non‐cell autonomous antioxidant support from astrocytes. Antioxidant defences, however, have been shown to significantly decrease during ageing in astrocytes themselves, with consequent accumulation of ROS (Ishii et al., 2017). A recent study showed that Nrf2 overexpression in astrocytes protects against cerebral hypoperfusion, thus suggesting that Nrf2 overexpression specifically in astrocytes is protective, exerting beneficial effects through repression of inflammation (Sigfridsson et al., 2018). In neurodegenerative diseases such as multiple sclerosis (MS), for example, astrocytes in active lesions display an increase of cytoplasmic accumulation of oxidised proteins and lipids (Horssen et al., 2008) and nuclear accumulation of oxidised DNA (Haider et al., 2011). As the antioxidant defences of astrocytes decrease with age, they are unable to provide optimal support to neighbouring neurons, which start accumulating oxidative DNA damage, thus leading to an increased susceptibility to a number of age‐related diseases and cognitive decline (Oksanen et al., 2019). Because of its crucial role in ageing and neurodegeneration, it is important that human in vitro models of astrocytes can recapitulate this age‐dependent functional decline.

Strikingly, in the present report, we found that old iNPC‐As exhibited a higher baseline level of ROS and DNA damage compared to young iNPC‐As. Furthermore, when iNPC‐As were stressed by serum starvation, old iNPC‐As required more time to recover to baseline ROS levels, as the levels of both NRF2 and SOD1 were lower in old iNPC‐As compared to young iNPC‐As, both at baseline and following acute stress. Thus, our model recapitulates the age‐related decrease in antioxidant defences reported from in vivo studies and provides an explanation for the increased level of ROS seen in these cells.

It is well known that oxidative stress plays a central role in the pathophysiology of neurodegenerative diseases, such as ALS, AD, HD and PD (Li et al., 2013; Niedzielska et al., 2016) where the antioxidant response has been proposed as a potential therapeutic target. Oxidative stress may provoke cellular damage, dysregulation of DNA damage repair and mitochondrial dysfunction, all hallmarks of the ageing process as well as neurodegenerative disorders (Allen et al., 2019; Shibata & Kobayashi, 2008).

Toxicity from free radicals also contributes to inflammation, another major component in neurodegeneration. In our study, we showed the age‐related differential responses of iNPC‐As in their ability to support neurons in co‐culture upon acute stimulation with the inflammatory cytokine interleukin‐1 beta (IL‐1β). Our data suggest that this is due to an exaggerated response of old donor‐derived iNPC‐As to IL‐1β stimulation, resulting in stronger nuclear activation of NFκΒ and higher levels of expression of GFAP and vimentin. Although already at baseline old and young iNPC‐As differ in the activation of several stress defence mechanisms, we did not detect a significant difference in their ability to support neuronal survival, measured as Casp3 activation. Other more subtle parameters, however, could be compromised in neurons cultured with older non stimulated astrocytes. Recent studies in post‐mortem brains and *C. elegans*, in fact, have reported that dendrite restructuring, neuronal sprouting, and synaptic deteriorations are subtle changes commonly detected in physiological brain ageing, rather than neurodegeneration (Henstridge et al., 2015; Hess et al., 2019).

GFAP and vimentin are classical astrocytic markers that increase in expression as astrocytes become more reactive with age (Boisvert et al., 2018; Clarke et al., 2018; Wu et al., 2005). Indeed, astrocytes have been described as going through activation stages ranging from mild to severe in response to physiological ageing or pathological and external insults, respectively. Mild reactivity is characterised by low levels of hypertrophy and expression of GFAP, while severe reactivity, for example in response to pro‐inflammatory stimuli, results in significant hypertrophy and GFAP expression increase (Sofroniew & Vinters, 2010). Our baseline and IL‐1b treatment data overall recapitulate these characteristics, where old iNPC‐As display larger area, as well as higher levels of GFAP and vimentin compared to young iNPC‐As at baseline. GFAP and vimentin levels then further increase upon IL‐1b treatment. In addition, increased astrocyte reactivity has been reported in several neurodegenerative disorders. For example, studies in AD brain samples and mouse models detected amyloid plaques surrounded by reactive astrocytes, with higher expression of GFAP (Li et al., 2011). Interestingly, the increased number of reactive astrocytes frequently correlates with the extent of cognitive decline (Kashon et al., 2004). In patients with PD, astrocyte reactivity has been seen in the substantia nigra pars compacta (Hirsch & Hunot, 2009), while in ALS patients, it has been observed in susceptible regions and the degree of reactivity correlates with the severity of neurodegeneration (Haim et al., 2015).

In conclusion, the present study demonstrates that direct conversion of fibroblasts to iNPCs and subsequent differentiation into iNPC‐As results in an in vitro model able to retain key hallmarks of the ageing process. Although not investigated in this study, it is likely that the iNPCs themselves and all cells derived from them would preserve ageing features through this reprogramming methodology. It is, in fact, unlikely that iNPCs would not carry ageing features and these would be gained in iNPC‐As is only 7 days of differentiation. This is of extreme importance, as this in vitro tool allows for more accurate and physiologically relevant modelling of ageing and disease, without the need to introduce age‐inducing genetic mutations or insults.

Interestingly, our results are in apparent contrast with the study by Sheng et al. (2018) who achieved direct reprogramming of peripheral blood cells (PBC) into iNPCs through a fast protocol (21 days). Consistent with our data, the authors observed retention of telomere length and a partial ageing signal in their transcriptomic study in iNPCs at low passage number. Both transcription profile and DNA methylation (DNAm) status, however, were largely affected by cell culture expansion and passaging >18 passages, which is our cut‐off for iNPC culture. Similarly, Sardo et al. (2017) reported loss of ageing features in iPSCs derived from donors aged 21–100 at passage 28 compared to 8. Although we did not assess the DNAm status of our iNPCs or iNPC‐As, our results would indicate that the ability of our reprogramming method to retain ageing features is likely to be related to several different aspects, including cells of origin, conversion efficiency, factors used during the conversion phase and, likely the most important factor, time in culture. PBCs present, in fact, several advantages for cell reprogramming, as an abundant source of cells; however, they are very susceptible to DNAm changes due to environmental factors and have intrinsically higher self‐renewal ability compared to fibroblasts (Ciccarone et al., 2018). These characteristics could contribute to the ability of the cells to retain ageing features after reprogramming. In addition, during PBC conversion to iNPCs, Sheng et al. (2018) selected iNPC colonies, thus introducing clonal selection. They also utilised a number of factors known to stimulate self‐renewal, such as CHIR, a WNT signalling activator, and LIF, a JAK‐STAT activator, involved in pluripotency maintenance in vitro (Onishi & Zandstra, 2015). As our transcriptomic study shows, WNT signalling downregulation is a feature of aged astrocytes, whether these are reprogrammed or from PM tissues, indicating that this pathway plays an important role in the ageing process. Hence, its upregulation might lead to rejuvenation processes likely to affect ageing feature retention. It would be interesting to test whether LIF or CHIR addition to our cultures would alter the ageing phenotype of the iNPCs and deriving cells.

Surprisingly, the use of the Yamanaka factors, that is, OKSM, did not affect the ability of our methodology to retain ageing features. In this context, Ocampo et al. (2016) and Sarkar et al. (2020) test the expression of the reprogramming factors only for a short ‘pulse’ and they both observe loss of ageing markers and de‐methylation, indicating that even short exposure to the Yamanaka factors is enough to cause loss of ageing. Interestingly, however, Ocampo et al report that, upon ‘switching off’ the expression of the transcription factors, some ageing markers are regained. One interesting hypothesis is that also in our protocol in the first 48 h the fibroblasts might experience a de‐methylation and rejuvenation wave, which enables the reprogramming, but that is then halted as iNPC differentiation starts, hence allowing for ageing epigenetic markers to be regained. Indeed, DNAm analysis of our iNPC cultures at different stages of conversion and differentiation would test this hypothesis and clarify the mechanisms involved in ageing preservation shown in this manuscript.

In summary, the ability of this model to recapitulate the behaviour of human astrocytes throughout the process of ageing as well as the crosstalk with age‐matched neurons has the unique and unprecedented potential to answer important questions about the physiology and the pathophysiology of the CNS and to provide a powerful tool for novel therapeutic approaches for age‐related neurodegenerative and neurological disorders.

## 4 EXPERIMENTAL PROCEDURES

### 4.1 Direct reprogramming of skin fibroblasts to iNPCs

Direct conversion was conducted with a modified version of the protocol described previously in Meyer et al. (2014). One lakh fibroblasts were seeded in a well of a six‐well plate and treated with retroviral vectors for *OCT3*, *SOX2*, *KLF4* and *c*‐*MYC* (Meyer et al., 2014). After 48 h, the medium was switched to NPC conversion medium, consisting of DMEM/F12, 1% N2, 1% B27, EGF (40 ng/ml) and FGF (20 ng/ml). Typically, after 2–4 days 60–80% of the fibroblasts changed shape to smaller morphology; some lifted and created sphere‐like structures, some started proliferating in rosettes or simply remained attached and changed morphology. These cells proliferated quickly and were split when 100% confluent. These were collected or lifted using Accutase (StemPro^®^ Accutase^®^ Cell Dissociation Reagent, Gibco) and, depending on the density and their growth rate, they were expanded in multiple wells of a six‐well plate or a 10‐cm dish coated with human fibronectin (5 μg/ml; Millipore) over a period of 18–21 days. After this first expansion period, during which the fibroblasts that did not reprogramme were diluted and eventually lost during iNPC expansion, cells were stained for Nestin and PAX6 to establish successful iNPC conversion. Typically, successful conversion is defined as yielding >95% cell positive for PAX6 and Nestin. This pure iNPC population was then expanded and stored for up to 18 passages. The lines used in this study all yielded a purity >98% regardless of the age of the donor (Figure S1).

### 4.2 Differentiation of iNPCs into iNPC‐As

To induce astrocyte differentiation, the iNPCs between passages 12–18 were plated in medium composed of DMEM, 10% foetal bovine serum (FBS) (Life Science Production), 1% Penicillin‐Streptomycin (Lonza Biowhittaker), 0.2% N2‐supplement (Gibco Life Tech) in a 10‐cm dish coated with human fibronectin (Millipore). Astrocytes were allowed to differentiate for 7 days. In all experiments, iNPCs from both young and old donor were passage matched within ±2 passages.

### 4.3 Differentiation of iPSCs into motor neurons (MNs)

Motor neuron differentiation (Figure S3) of iPSCs was performed using the modified version dual SMAD inhibition protocol (Du et al., 2015). This protocol typically yields ~90% ChAT^+^ MNs.

### 4.4 Co‐culture of iNPC‐As and MNs

Motor neuron progenitors were plated at a density of 1 × 10^4^ cell/well onto matrigel‐coated 96‐well plates in the presence of 10 µM rock inhibitor on day 20 of differentiation. The MNPs were differentiated into MN until day 40 when the astrocytes were added on the top of the motor neurons at a density of 0.8 × 10^4^ cell/well. The co‐cultures were cultured for three days, and then, the cells were fixed and processed for immunofluorescence staining (Figure S4).

### 4.5 Microarray analysis

Microarray analysis of human foetal astrocytes (purchased from ScienCell; pool of foetal astrocytes isolated from human brain cortex), PM astrocytes laser‐captured from human brain and iNPC‐As reprogrammed from fibroblast donors was performed at SITraN, University of Sheffield (Table S2). Foetal astrocytes were run on GeneChip™ Human Gene 2.0 ST Array, while postmortem astrocytes and iNPC‐As were run on GeneChip™ Human Genome U133 Plus 2.0 Array. All the data were already available at the beginning of the study and all had obtained relevant ethical approval. The quality of array data CEL files was assessed using the Affymetrix Expression Console software. Normalisation, with all the transcripts normalised by beta‐tubulin and GAPDH, and harmonisation across Chip files was obtained using the Robust Multi‐Array (RMA) algorithm. Normalised data have been submitted as a Pivot Table to the Online Research Data (ORDA) repository (link: 10.15131/shef.data.12162282). Differential gene expression between samples was determined using Qlucore Omics Explorer after normalisation. The software was used also for Principal Component Analysis (PCA) and hierarchical clustering. To access the functions of genes found in the meta‐analysis, the Database for Annotation, Visualisation and Integrated Discovery (DAVID, https://david.ncifcrf.gov/) and Panther (http://www.pantherdb.org/). The website Human Genomic Resources (HAGR, http://genomics.senescence.info/) was used to identify ageing‐related genes.

### 4.6 RNA Isolation and quantitative real‐time polymerase chain reaction (qPCR)

RNA was harvested using the RNAeasy Mini kit (Qiagen), and total RNA was reverse‐transcribed with the High Capacity cDNA Reverse Kit (Applied Biosystem) according to the manufacturer's instructions.

Primers were designed using Primer‐BLAST to assess transcriptional changes in a number of selected genes reported by microarray analysis identified through HAGR and validated ageing markers (Table S4). Samples were loaded at a concentration of 12.5 ng/μl per well. qPCR was performed using 2x SYBR Green/Rox PCR Master Mix (Bimake.com) and forward and reverse primers (final concentration 250 nM), to a total volume of 20 µl. After initial denaturation at 95°C for 10 min, templates were amplified by 40 cycles at 95°C for 15 s and 60°C for 1 min, using the Stratagene MX300P machine. At the end, a dissociation curve was created to ensure amplification of a single product and the absence of primer dimers.

GAPDH was tested against other housekeeping genes, including B‐actin, RLP13A and U1. GAPDH showed the most consistent and stable expression across samples of different ages as well as between fibroblasts and astrocytes. Hence, GAPDH was amplified on each plate to normalise expression levels of target genes between different samples using the ΔΔCt calculation (ABI) and to assess assay reproducibility.

### 4.7 Telomere length measurement

DNA from iPSC‐ and iNPC‐derived young and old astrocytes was extracted with the DNeasy Blood & Tissue Kit (QIAGEN) according to the manufacturer's protocol. Telomere length of iPSC and iNPC‐derived astrocytes was measured using the Absolute Human Telomere Length quantification qPCR assay Kit (ScienCell Research Laboratories, San Diego, CA, USA) according to the manufacturer's instructions. A single copy reference (SCR) primer set recognises and amplifies a 100‐bp‐long region on human chromosome 17 and is used as a reference for data normalisation. The reference genomic DNA sample with known telomere length was used as reference for calculating the Telomere Length of iPSC (Table S5) and young and old iNPC‐derived astrocytes. qPCR products were performed on a C1000 Touch™ thermos Cycler CFX96™ Real‐Time System (Bio‐Rad), and qPCR data were analysed using CFX Manager™ software (Version 3.1) (Bio‐Rad) and GraphPad Prism (Version 8).

### 4.8 iNPC‐A treatment for stress response assessment and cell collection

Serum starvation experiments were carried out by washing the cells with PBS twice and changing the culture medium to fresh media without FBS at day 6.

On day 7, iNPC‐As were washed twice with PBS to remove medium residuals and harvested by cell scraping. The cell pellets were stored at −80°C until processed for RNA or protein extraction.

### 4.9 IL‐1β treatment to induce inflammation in iNPC‐As and CC

Astrocyte activation was stimulated by the addition of fresh medium containing recombinant human IL‐1β (20 ng/ml, R&D Systems) and incubated for 20 min for evaluation of NF‐κB translocation, for 24 and 72 h and for reactive gliosis.

For the co‐culture experiments, young and old astrocytes were stimulated with recombinant human IL‐1β (20 ng/ml R&D Systems), for 6 h and then gently washed three times with warm PBS to fully eliminate residual IL‐1β in the culture. After that the astrocytes were seeded on the top of the MNs in the presence of neuronal medium. The co‐cultures were maintained at 37°C in a humidified atmosphere with 5% CO_2_ for 3 days when the analyses of cell death was performed. Young and old untreated astrocytes were used as controls.

After the treatment, the cells were fixed and stained for vimentin as a astrocytic marker, active caspase three to detect apoptotic cells, MAP2 (neuronal marker) was used as a marker that define the boundary of cells and DAPI for nuclear staining. A quantitative imaging analysis of the apoptotic neurons was conducted through the Opera Phenix™ High Content Screening System at ×40 magnification using the Harmony High‐Content Imaging and Analysis Software. Quantification of apoptotic neurons was assessed for both treated and untreated cells, whereby only cells that stained positive for MAP2 were evaluated for caspase 3 staining. Twenty fields per well of a 96‐well plate were randomly selected, scanned and staining quantified, thus leading to the quantification of about 100 cells/well. Each co‐culture condition was run in triplicate (technical repeats).

### 4.10 Immunocytochemistry (ICC) iNPC‐As

Ten thousand iNPC‐As were plated per well in 96‐well plates at day 6 of differentiation and fixed 24 h after seeding with 4% paraformaldehyde (PFA) for 10 min. Cells were washed three times with phosphate‐buffered saline (PBS) following which blocking (PBS, 5% horse serum and 0.05% Triton X‐100) was applied for 1 h. All primary antibodies were diluted in blocking solution and their dilution and suppliers are listed in Table S6. Incubation of the primary antibody was performed overnight at 4°C. The next day, cells were washed three times in PBS before the secondary antibody (Table S7) in blocking solution was applied for 1 h at room temperature. Hoechst (Hoechst 33342, Trihydrochloride, Trihydrate, Life tech) diluted 1:6000 was added for 5 min to visualise the nucleus. Cells were then washed twice with PBS and imaged using the Opera Phenix high‐content imager (PerkinElmer).

### 4.11 Immunocytochemistry (ICC) MNs and CC

For immunostaining, after three days of co‐culture (CC), cells were washed with PBS and fixed with 4% paraformaldehyde for 15 min at room temperature. After fixation samples were washed three times with PBS, incubated with 0.3% Triton X‐100 in PBS (Sigma) for 5 min then blocked by incubation with PBS containing 5% donkey serum (DS) (Millipore) for 1 h. After blocking, cell cultures were incubated with primary antibodies diluted in PBS containing 1% of DS overnight. Cells were washed with PBS three times. Then, secondary antibodies were added to cells and incubated for 1 h. The samples were washed with PBS three more times and incubated with 1.0 mg/ml 4,6‐diamidino‐2‐phenylindole (DAPI) for nuclear staining. The primary and secondary antibodies were used at the dilutions indicated in tables S6 and S7. All experiments included cultures where the primary antibodies were not added, and non‐specific staining was not observed in these negative controls.

### 4.12 Oxidative stress assay

Seven thousand iNPC‐As were plated per well in 96‐well plates at day 5 of differentiation in complete astrocyte medium. On day 6, medium was replaced either with complete or with serum‐free astrocyte medium for 12 hours or with complete medium plus 0.0016% V/V β‐mercaptoethanol as a negative control. After 12 h, medium was replaced in all conditions with complete astrocyte medium and CellROX baseline measurements were collected, using the Opera Phenix Imaging System. A time‐course experiment was also performed, in which CellROX measurements were collected every 2 h for 6 h. CellROX^®^ Reagent (Life Technologies) was used at a final concentration of 2.5 μM as per manufacturer's instructions and incubated with cells for 30 min at 37°C. CellROX^®^ Reagent was then removed, and cells were washed three times with PBS before imaging.

### 4.13 Nuclear isolation and dextran influx assay

Two 10‐cm dishes of iNPC‐As (two million cells) were used for nuclei isolation. After two washes with PBS, accutase was added and the plates were incubated at 37°C for 3 min. Accutase was diluted with 3 ml PBS, and the cells were collected in a tube and spun at 200 *g* for 4 min. The pellet was washed twice with PBS and then left to dry. 400 µl of hypotonic lysis buffer (10 mM HEPES, 1.5 mM MgCl2, 10 mM KCl, 0.5 mM DTT, 20 µl/ml PIC) was added to the pellet. The lysate was passed through a 19 g needle five times, left for 10 min on ice and then spun down at 800 *g* for 3 min at 4°C to separate the nuclear fraction from the cytoplasmic. The supernatant was discarded (cytoplasmic fraction), while 250 µl of IP lysis buffer (150 mM NaCl, 50 mM HEPES, 1 mM EDTA, 1 mM DTT, 0.5% (v/v) Triton™ X‐100, 20 µl/ml PIC, pH 8.0) with 60 µl of PMSF (phenylmethanesulfonyl) was added to the nuclear pellet. The lysate was passed through a 25 g needle five times and then left for 30 min on ice. It was spun at 10,000 *g* for four minutes at 4°C, and the supernatant was kept as the nuclear fraction. Nuclei were stained with Hoechst (Hoechst 33342, Trihydrochloride, Trihydrate, Life tech) diluted 1:6000 in PBS. Tetramethylrhodamine isothiocyanate‐dextran was added to the nuclear fraction. The nuclei were mounted with ProLong™ Gold Antifade Mountant (Invitrogen) on cover slips and imaged with the confocal microscope Leica TCS SP5 II.

### 4.14 Western Blot (WB) Analysis

iNPC‐A samples were lysed using lysis buffer (150 mM NaCl, 50 mM HEPES, 1 mM EDTA, 1 mM DTT, 0.5% (v/v) Triton™ X‐100, PIC, pH 8.0). Samples were then centrifuged at 13,300 rpm for 5 min at 4°C, and the supernatant was collected. Lysates were subjected to sodium dodecyl sulphate‐polyacrylamide gel electrophoresis (SDS‐PAGE) on 12% polyacrylamide gels. Resolved proteins were transferred to nitrocellulose membranes and processed for immunoblot analysis.

Membranes were blocked for 1 h in 5% milk/tris‐buffered saline and Tween‐20 (TBS‐T). Primary antibodies (Table S6) were diluted in 5% milk/TBS‐T and incubated with the membrane overnight at 4°C. After primary antibody incubation, the membrane was washed for 3 × 5 min each in TBS‐T buffer. Secondary horseradish peroxidase (HRP) antibodies (Table S7) were diluted in 5% milk/TBS‐T and incubated with the membrane for 1 h at room temperature. After secondary antibody incubation, the membrane was washed for 3 × 5 min each in TBS‐T buffer. Protein bands were visualised using EZ‐ECL reagent (Geneflow) and the G‐Box imaging system (Syngene). Densitometric analysis of protein bands was carried out using GeneTools Software (Syngene).

### 4.15 Image and statistical analysis

Columbus Software was used in the image analysis setting to measure different parameters including nuclei numbers, nuclear size (μm^2^) (mean per well), CellROX intensity/Cell area, Lamin A/C as nuclear roundness (arbitrary units = AU) and H3K9me3 intensity (arbitrary fluorescent units = AFU).

To investigate differences in nuclear envelope morphology, iNPC‐As nuclei were first detected using DAPI with boundary objects removed. The nuclear envelope was detected by expression of Lamin A/C restricted to the perinuclear space. This enabled assessment of blebs or irregularities that expand within the nuclear DAPI signal, which correlated with a decrease in nuclear envelope roundness. Data were visualised as individual cell nuclear envelope roundness using a violin plot with an upper limit of 0.3, which was greater than the 95% percentile for both groups.

To investigate whether the IL‐1β interferes with the translocation of NF‐κB, young and old astrocytes were stained for NF‐κB, untreated cells were used as a control for each line. Reactive gliosis was determined by GFAP and Vim staining intensity (AFU), which was quantified using Harmony software (Perkin Elmer). After the treatment the cells were fixed and stained for NF‐κB and vimentin was used as a marker that defines the boundary of cells and DAPI for nuclear staining. Quantitative image analysis of the astrocytes was conducted through the Opera Phenix™ High Content Screening System at 40× magnification using the Columbus™ Image analysis system. The following morphological features were assessed for both treated and control astrocytes: cell area and the intensity of GFAP and vimentin staining per cell. At least 20 fields were randomly selected and scanned per well of a 96‐well plate in triplicate. To identify and remove any false readings generated by the system, three random treated and untreated wells were selected and counted manually (blind to group).

All experiments were performed at least in triplicate (i.e., three independent experimental repeats from three different iNPC‐A differentiation rounds) with a minimum of three technical repeats in each independent experiment. Data were analysed using GraphPad Prism Software (V7.02): one‐way ANOVA with Tukey's multiple comparisons post‐test or two‐way ANOVA with Tukey's multiple comparisons post‐test. Two‐tailed *t* test was used to compare old donor samples to the young donor samples for qPCR and Western blot.

## CONFLICT OF INTEREST

The authors declare no competing interests.

## AUTHOR CONTRIBUTION

LF conceptualised the study; NG, CDS, SMB, AS, MAM, SP, LC, EK and NMM performed the experiments; NG, NMM and LF wrote original draft; all authors reviewed and edited the manuscript; LF and PJS secured the funding.

## Supporting information

Supinfo

TableS3

## Data Availability

The data that support the findings of this study are openly available in GEO and ORDA at https://www.ncbi.nlm.nih.gov/geo/ and https://orda.shef.ac.uk/, reference number indicated in the manuscript for each dataset.

## References

[acel13281-bib-0001] Allen, S. P. , Hall, B. , Woof, R. , Francis, L. , Gatto, N. , Shaw, A. C. , Myszczynska, M. , Hemingway, J. , Coldicott, I. , Willcock, A. , Job, L. , Hughes, R. M. , Boschian, C. , Bayatti, N. , Heath, P. R. , Bandmann, O. , Mortiboys, H. , Ferraiuolo, L. , & Shaw, P. J. (2019). C9orf72 expansion within astrocytes reduces metabolic flexibility in amyotrophic lateral sclerosis. Brain: a Journal of Neurology, 142(12), 3771–3790. 10.1093/brain/awz302 31647549 PMC6906594

[acel13281-bib-0002] Almad, A. , & Maragakis, N. J. (2018). A stocked toolbox for understanding the role of astrocytes in disease. Nature Reviews Neurology, 14(6), 351–362. 10.1038/s41582-018-0010-2 29769699

[acel13281-bib-0003] Bell, K. F. , Al‐Mubarak, B. , Fowler, J. H. , Baxter, P. S. , Gupta, K. , Tsujita, T. , Chowdhry, S. , Patani, R. , Chandran, S. , Horsburgh, K. , Hayes, J. D. , & Hardingham, G. E. (2011). Mild oxidative stress activates Nrf2 in astrocytes, which contributes to neuroprotective ischemic preconditioning. Proceedings of the National Academy of Sciences, 108(1), E1–E2. 10.1073/pnas.1015229108 PMC3017195 21177433

[acel13281-bib-0004] Bell, K. F. S. , Al‐Mubarak, B. , Martel, M.‐A. , McKay, S. , Wheelan, N. , Hasel, P. , Márkus, N. M. , Baxter, P. , Deighton, R. F. , Serio, A. , Bilican, B. , Chowdhry, S. , Meakin, P. J. , Ashford, M. L. J. , Wyllie, D. J. A. , Scannevin, R. H. , Chandran, S. , Hayes, J. D. , & Hardingham, G. E. (2015). Neuronal development is promoted by weakened intrinsic antioxidant defences due to epigenetic repression of Nrf2. Nature Communications, 6(1). 10.1038/ncomms8066 PMC4441249 25967870

[acel13281-bib-0005] Bellaver, B. , Souza, D. G. , Souza, D. O. , & Quincozes‐Santos, A. (2017). Hippocampal astrocyte cultures from adult and aged rats reproduce changes in glial functionality observed in the aging brain. Molecular Neurobiology, 54(4), 2969–2985. 10.1007/s12035-016-9880-8 27026184

[acel13281-bib-0006] Ben Haim, L. , Carrillo‐de Sauvage, M.‐A. , CeyzÃ©riat, K. , & Escartin, C. (2015). Elusive roles for reactive astrocytes in neurodegenerative diseases. Frontiers in Cellular Neuroscience, 9 10.3389/fncel.2015.00278 PMC4522610 26283915

[acel13281-bib-0007] Blasko, I. , Veerhuis, R. , Stampfer‐Kountchev, M. , Saurwein‐Teissl, M. , Eikelenboom, P. , & Grubeck‐Loebenstein, B. (2000). Costimulatory effects of interferon‐β and interleukin‐1β or tumor necrosis factor α on the synthesis of Aβ1‐40 and Aβ1‐42 by human astrocytes. Neurobiology of Disease, 7(6), 682–689. 10.1006/nbdi.2000.0321 11114266

[acel13281-bib-0008] Boczonadi, V. , Meyer, K. , Gonczarowska‐Jorge, H. , Griffin, H. , Roos, A. , Bartsakoulia, M. , Bansagi, B. , Ricci, G. , Palinkas, F. , Zahedi, R. P. , Bruni, F. , Kaspar, B. , Lochmüller, H. , Boycott, K. M. , Müller, J. S. , & Horvath, R. (2018). Mutations in glycyl‐tRNA synthetase impair mitochondrial metabolism in neurons. Human Molecular Genetics, 27(12), 2187–2204. 10.1093/hmg/ddy127 29648643 PMC5985729

[acel13281-bib-0009] Boisvert, M. M. , Erikson, G. A. , Shokhirev, M. N. , & Allen, N. J. (2018). The aging astrocyte transcriptome from multiple regions of the mouse brain. Cell Reports, 22(1), 269–285. 10.1016/j.celrep.2017.12.039 29298427 PMC5783200

[acel13281-bib-0010] Booth, H. D. E. , Hirst, W. D. , & Wade‐Martins, R. (2017). Role of astrocyte dysfunction in Parkinson’s disease pathogenesis. Trends in Neurosciences, 40(6), 358–370. 10.1016/j.tins.2017.04.001 28527591 PMC5462417

[acel13281-bib-0011] Brawek, B. , Löffler, M. , Wagner, K. , Huppertz, H.‐J. , Wendling, A.‐S. , Weyerbrock, A. , Jackisch, R. , & Feuerstein, T. J. (2010). Reactive oxygen species (ROS) in the human neocortex: Role of aging and cognition. Brain Research Bulletin, 81(4–5), 484–490. 10.1016/J.BRAINRESBULL.2009.10.011 19854245

[acel13281-bib-0012] Canals, I. et al (2018). Rapid and efficient induction of functional astrocytes from human pluripotent stem cells. Nature Methods, 16(1), 134 10.1038/s41592-018-0264-z 30514884

[acel13281-bib-0013] Chai, M. , & Kohyama, J. (2019). Non‐Cell‐Autonomous Neurotoxicity in Parkinson’s Disease Mediated by Astroglial α‐Synuclein. Stem Cell Reports, 12(2), 183–185. 10.1016/j.stemcr.2019.01.011 30759377 PMC6373431

[acel13281-bib-0014] Chan, W. M. , Tsoi, H. O. , Wu, C. C. , Wong, C. H. , Cheng, T. C. , Li, H. Y. , Lau, K. F. , Shaw, P. C. , Perrimon, N. , & Chan, H. Y. E. (2011). Expanded polyglutamine domain possesses nuclear export activity which modulates subcellular localization and toxicity of polyQ disease protein via exportin‐1. Human Molecular Genetics, 20(9), 1738–1750. 10.1093/hmg/ddr049 21300695

[acel13281-bib-0015] Ciccarone, F. , Tagliatesta, S. , Caiafa, P. , & Zampieri, M. (2018). DNA methylation dynamics in aging: how far are we from understanding the mechanisms? Mechanisms of Ageing and Development, 174, 3–17. 10.1016/j.mad.2017.12.002 29268958

[acel13281-bib-0016] Clarke, L. E. , Liddelow, S. A. , Chakraborty, C. , Münch, A. E. , Heiman, M. , & Barres, B. A. (2018) Normal aging induces A1‐like astrocyte reactivity. Proceedings of the National Academy of Sciences, 115(8), E1896–E1905. 10.1073/pnas.1800165115 PMC5828643 29437957

[acel13281-bib-0017] D'Angelo, M. A. , Raices, M. , Panowski, S. H. , & Hetzer, M. W. (2009). Age‐dependent deterioration of nuclear pore complexes causes a loss of nuclear integrity in postmitotic cells. Cell, 136(2), 284–295. 10.1016/j.cell.2008.11.037 19167330 PMC2805151

[acel13281-bib-0018] di Domenico, A. , Carola, G. , Calatayud, C. , Pons‐Espinal, M. , Muñoz, J. P. , Richaud‐Patin, Y. , Fernandez‐Carasa, I. , Gut, M. , Faella, A. , Parameswaran, J. , Soriano, J. , Ferrer, I. , Tolosa, E. , Zorzano, A. , Cuervo, A. M. , Raya, A. , & Consiglio, A. (2019). Patient‐specific iPSC‐derived astrocytes contribute to non‐cell‐autonomous neurodegeneration in parkinson’s disease. Stem Cell Reports, 12(2), 213–229. 10.1016/j.stemcr.2018.12.011 30639209 PMC6372974

[acel13281-bib-0019] Dong, X. , Biswas, A. , Süel, K. E. , Jackson, L. K. , Martinez, R. , Gu, H. , & Chook, Y. M. (2009). Structural basis for leucine‐rich nuclear export signal recognition by CRM1. Nature, 458(7242), 1136–1141. 10.1038/nature07975 19339969 PMC3437623

[acel13281-bib-0020] Du, Z.‐W. , Chen, H. , Liu, H. , Lu, J. , Qian, K. , Huang, C.‐L. , Zhong, X. , Fan, F. , & Zhang, S.‐C. (2015). Generation and expansion of highly pure motor neuron progenitors from human pluripotent stem cells. Nature Communications, 6(1). 10.1038/ncomms7626 PMC4375778 25806427

[acel13281-bib-0021] Duan, W. , Zhang, R. , Guo, Y. , Jiang, Y. , Huang, Y. , Jiang, H. , & Li, C. (2009). Nrf2 activity is lost in the spinal cord and its astrocytes of aged mice. In Vitro Cellular & Developmental Biology ‐ Animal, 45(7), 388–397. 10.1007/s11626-009-9194-5 19452231

[acel13281-bib-0022] Ederle, H. , Funk, C. , Abou‐Ajram, C. , Hutten, S. , Funk, E. B. E. , Kehlenbach, R. H. , Bailer, S. M. , & Dormann, D. (2018). Nuclear egress of TDP‐43 and FUS occurs independently of Exportin‐1/CRM1. Scientific Reports, 8(1), 1–18. 10.1038/s41598-018-25007-5 29728564 PMC5935713

[acel13281-bib-0023] Eftekharzadeh, B. , Daigle, J. G. , Kapinos, L. E. , Coyne, A. , Schiantarelli, J. , Carlomagno, Y. , Cook, C. , Miller, S. J. , Dujardin, S. , Amaral, A. S. , Grima, J. C. , Bennett, R. E. , Tepper, K. , DeTure, M. , Vanderburg, C. R. , Corjuc, B. T. , DeVos, S. L. , Gonzalez, J. A. , Chew, J. , … Hyman, B. T. (2018). ‘Tau Protein Disrupts Nucleocytoplasmic Transport in Alzheimer’s Disease’., *Neuron*. NIH Public. Access, 99(5), 925–940.e7. 10.1016/j.neuron.2018.07.039 PMC6240334 30189209

[acel13281-bib-0024] Fasching, C. L. (2018). Telomere length measurement as a clinical biomarker of aging and disease. Critical Reviews in Clinical Laboratory Sciences, 55(7), 443–465. 10.1080/10408363.2018.1504274 30265166

[acel13281-bib-0025] Ferraiuolo, L. , Meyer, K. , Sherwood, T. W. , Vick, J. , Likhite, S. , Frakes, A. , Miranda, C. J. , Braun, L. , Heath, P. R. , Pineda, R. , Beattie, C. E. , Shaw, P. J. , Askwith, C. C. , McTigue, D. , & Kaspar, B. K. (2016). Oligodendrocytes contribute to motor neuron death in ALS via SOD1‐dependent mechanism. Proceedings of the National Academy of Sciences, 113(42), E6496–E6505. 10.1073/pnas.1607496113 PMC5081600 27688759

[acel13281-bib-0026] Ferreira, P. A. (2019). The coming‐of‐age of nucleocytoplasmic transport in motor neuron disease and neurodegeneration. Cellular and Molecular Life Sciences, 76(12), 2247–2273. 10.1007/s00018-019-03029-0 30742233 PMC6531325

[acel13281-bib-0027] Freibaum, B. D. , Lu, Y. , Lopez‐Gonzalez, R. , Kim, N. C. , Almeida, S. , Lee, K.‐H. , Badders, N. , Valentine, M. , Miller, B. L. , Wong, P. C. , Petrucelli, L. , Kim, H. J. , Gao, F.‐B. , & Taylor, J. P. (2015). GGGGCC repeat expansion in C9orf72 compromises nucleocytoplasmic transport. Nature, 525(7567), 129–133, 10.1038/nature14974 26308899 PMC4631399

[acel13281-bib-0028] Gasset‐Rosa, F. , Chillon‐Marinas, C. , Goginashvili, A. , Atwal, R. S. , Artates, J. W. , Tabet, R. , Wheeler, V. C. , Bang, A. G. , Cleveland, D. W. , & Lagier‐Tourenne, C. (2017). Polyglutamine‐expanded huntingtin exacerbates age‐related disruption of nuclear integrity and nucleocytoplasmic transport. Neuron, 94(1), 48–57. e4. 10.1016/j.neuron.2017.03.027 28384474 PMC5479704

[acel13281-bib-0029] Gemma, C. , Vila, J. , Bachstetter, A. , & Bickford, P. C. (2007). Oxidative stress and the aging brain: from theory to prevention, brain aging: models, methods, and mechanisms. Retrieved from http://www.ncbi.nlm.nih.gov/pubmed/21204345 21204345

[acel13281-bib-0030] Ghosh, S. , & Zhou, Z. (2014). Genetics of aging, progeria and lamin disorders. Current Opinion in Genetics and Development, 26, 41–46. 10.1016/j.gde.2014.05.003 25005744

[acel13281-bib-0031] Godin, L. M. , Sandri, B. J. , Wagner, D. E. , Meyer, C. M. , Price, A. P. , Akinnola, I. , Weiss, D. J. , & Panoskaltsis‐Mortari, A. (2016). Decreased laminin expression by human lung epithelial cells and fibroblasts cultured in acellular lung scaffolds from aged mice. PLoS One, 11(3), e0150966 10.1371/journal.pone.0150966 26954258 PMC4783067

[acel13281-bib-0032] González‐Reyes, R. E. , Nava‐Mesa, M. O. , Vargas‐Sánchez, K. , Ariza‐Salamanca, D. , & Mora‐Muñoz, L. (2017). Involvement of astrocytes in Alzheimer’s disease from a neuroinflammatory and oxidative stress perspective. Frontiers in Molecular Neuroscience, 10, 427 10.3389/fnmol.2017.00427 29311817 PMC5742194

[acel13281-bib-0033] Gonzalo, S. , Kreienkamp, R. , & Askjaer, P. (2017). Hutchinson‐Gilford Progeria Syndrome: A premature aging disease caused by LMNA gene mutations. Ageing Research Reviews, 33, 18–29. 10.1016/j.arr.2016.06.007 27374873 PMC5195863

[acel13281-bib-0034] Grima, J. C. , Daigle, J. G. , Arbez, N. , Cunningham, K. C. , Zhang, K. E. , Ochaba, J. , Geater, C. , Morozko, E. , Stocksdale, J. , Glatzer, J. C. , Pham, J. T. , Ahmed, I. , Peng, Q. I. , Wadhwa, H. , Pletnikova, O. , Troncoso, J. C. , Duan, W. , Snyder, S. H. , Ranum, L. P. W. , … Rothstein, J. D. (2017). Mutant huntingtin disrupts the nuclear pore complex. Neuron, 94(1), 93–107. e6. 10.1016/j.neuron.2017.03.023 28384479 PMC5595097

[acel13281-bib-0035] Haider, L. , Fischer, M. T. , Frischer, J. M. , Bauer, J. , Hoftberger, R. , Botond, G. , Esterbauer, H. , Binder, C. J. , Witztum, J. L. , & Lassmann, H. (2011). Oxidative damage in multiple sclerosis lesions. Brain, 134(7), 1914–1924. 10.1093/brain/awr128 21653539 PMC3122372

[acel13281-bib-0036] Haidet‐Phillips, A. M. , Hester, M. E. , Miranda, C. J. , Meyer, K. , Braun, L. , Frakes, A. , Song, S. W. , Likhite, S. , Murtha, M. J. , Foust, K. D. , Rao, M. , Eagle, A. , Kammesheidt, A. , Christensen, A. , Mendell, J. R. , Burghes, A. H. M. , & Kaspar, B. K. (2011). Astrocytes from familial and sporadic ALS patients are toxic to motor neurons. Nature Biotechnology, 29(9), 824–828. 10.1038/nbt.1957 PMC3170425 21832997

[acel13281-bib-0037] Haithcock, E. , Dayani, Y. , Neufeld, E. , Zahand, A. J. , Feinstein, N. , Mattout, A. , Gruenbaum, Y. , & Liu, J. (2005). Age‐related changes of nuclear architecture in Caenorhabditis elegans. Retrieved from www.pnas.orgcgidoi10.1073pnas.0506955102 10.1073/pnas.0506955102 PMC1283819 16269543

[acel13281-bib-0038] Hautbergue, G. M. , Castelli, L. M. , Ferraiuolo, L. , Sanchez‐Martinez, A. , Cooper‐Knock, J. , Higginbottom, A. , Lin, Y.‐H. , Bauer, C. S. , Dodd, J. E. , Myszczynska, M. A. , Alam, S. M. , Garneret, P. , Chandran, J. S. , Karyka, E. , Stopford, M. J. , Smith, E. F. , Kirby, J. , Meyer, K. , Kaspar, B. K. , … Shaw, P. J. (2017). SRSF1‐dependent nuclear export inhibition of C9ORF72 repeat transcripts prevents neurodegeneration and associated motor deficits. Nature Communications, 8(1). 10.1038/ncomms16063 PMC5504286 28677678

[acel13281-bib-0039] Henstridge, C. M. , Jackson, R. J. , Kim, J. S. M. , Herrmann, A. G. , Wright, A. K. , Harris, S. E. , Bastin, M. E. , Starr, J. M. , Wardlaw, J. , Gillingwater, T. H. , Smith, C. , McKenzie, C.‐A. , Cox, S. R. , Deary, I. J. , & Spires‐Jones, T. L. (2015). Post‐mortem brain analyses of the Lothian Birth Cohort 1936: Extending lifetime cognitive and brain phenotyping to the level of the synapse. Acta Neuropathologica Communications, 3(1). 10.1186/s40478-015-0232-0 PMC4559320 26335101

[acel13281-bib-0040] Hess, M. , Gomariz, A. , Goksel, O. , & Ewald, C. Y. (2019) In‐vivo quantitative image analysis of age‐related morphological changes of C. elegans neurons reveals a correlation between neurite bending and novel neurite outgrowths. eNeuro, 6(4). 10.1523/ENEURO.0014-19.2019 PMC6620389 31217194

[acel13281-bib-0041] Hirsch, E. C. , & Hunot, S. (2009). Neuroinflammation in Parkinson’s disease: A target for neuroprotection? The Lancet Neurology, 382–397. 10.1016/S1474-4422(09)70062-6 19296921

[acel13281-bib-0042] Huh, C. J. , Zhang, B. O. , Victor, M. B. , Dahiya, S. , Batista, L. F. Z. , Horvath, S. , & Yoo, A. S. (2016). Maintenance of age in human neurons generated by microRNA‐based neuronal conversion of fibroblasts. eLife, 5 10.7554/eLife.18648 PMC5067114 27644593

[acel13281-bib-0043] Hutten, S. , & Dormann, D. (2019). Nucleocytoplasmic transport defects in neurodegeneration — Cause or consequence? Seminars in Cell & Developmental Biology, 99, 151–162. 10.1016/J.SEMCDB.2019.05.020 31152789

[acel13281-bib-0044] Ishii, T. , Takanashi, Y. , Sugita, K. , Miyazawa, M. , Yanagihara, R. , Yasuda, K. , Onouchi, H. , Kawabe, N. , Nakata, M. , Yamamoto, Y. , Hartman, P. S. , & Ishii, N. (2017). Endogenous reactive oxygen species cause astrocyte defects and neuronal dysfunctions in the hippocampus: A new model for aging brain. Aging Cell, 16(1), 39–51. 10.1111/acel.12523 27623715 PMC5242301

[acel13281-bib-0045] Jovičić, A. , Mertens, J. , Boeynaems, S. , Bogaert, E. , Chai, N. , Yamada, S. B. , Paul, J. W. , Sun, S. , Herdy, J. R. , Bieri, G. , Kramer, N. J. , Gage, F. H. , Van Den Bosch, L. , Robberecht, W. , & Gitler, A. D. (2015). Modifiers of C9orf72 dipeptide repeat toxicity connect nucleocytoplasmic transport defects to FTD/ALS. Nature Neuroscience. Nature Publishing Group, 18(9), 1226–1229. 10.1038/nn.4085 PMC4552077 26308983

[acel13281-bib-0046] Kashon, M. L. , Ross, G. W. , O'Callaghan, J. P. , Miller, D. B. , Petrovitch, H. , Burchfiel, C. M. , Sharp, D. S. , Markesbery, W. R. , Davis, D. G. , Hardman, J. , Nelson, J. , & White, L. R. (2004). Associations of cortical astrogliosis with cognitive performance and dementia status. Journal of Alzheimer’s Disease, 6(6), 595–604. 10.3233/jad-2004-6604 15665400

[acel13281-bib-0047] Kim, H. J. , & Taylor, J. P. (2017). Lost in transportation: nucleocytoplasmic transport defects in ALS and other neurodegenerative diseases. Neuron. NIH Public Access, 96(2), 285–297. 10.1016/j.neuron.2017.07.029 PMC5678982 29024655

[acel13281-bib-0048] Koch, P. , Bohlmann, I. , Schäfer, M. , Hansen‐Hagge, T. E. , Kiyoi, H. , Wilda, M. , Hameister, H. , Bartram, C. R. , & Janssen, J. W. G. (2000). Identification of a novel putative Ran‐binding protein and its close homologue. Biochemical and Biophysical Research Communications, 278(1), 241–249. 10.1006/bbrc.2000.3788 11071879

[acel13281-bib-0049] Lapasset, L. , Milhavet, O. , Prieur, A. , Besnard, E. , Babled, A. , Ait‐Hamou, N. , Leschik, J. , Pellestor, F. , Ramirez, J.‐M. , De Vos, J. , Lehmann, S. , & Lemaitre, J.‐M. (2011). Rejuvenating senescent and centenarian human cells by reprogramming through the pluripotent state. Genes & Development, 25(21), 2248–2253. 10.1101/gad.173922.111 22056670 PMC3219229

[acel13281-bib-0050] Lee, J. , Hyeon, S. J. , Im, H. , Ryu, H. , Kim, Y. , & Ryu, H. (2016). Astrocytes and microglia as non‐cell autonomous players in the pathogenesis of ALS. Experimental Neurobiology, 25(5), 233–240. 10.5607/en.2016.25.5.233 27790057 PMC5081469

[acel13281-bib-0051] Lee, J. H. , Zhou, S. , & Smas, C. M. (2010). Identification of RANBP16 and RANBP17 as novel interaction partners for the bHLH transcription factor E12. Journal of Cellular Biochemistry, 111(1), 195–206. 10.1002/jcb.22689 20503194 PMC2930062

[acel13281-bib-0052] Lénárt, P. , & Ellenberg, J. (2006). Monitoring the permeability of the nuclear envelope during the cell cycle. Methods, 38(1), 17–24. 10.1016/j.ymeth.2005.07.010 16343937

[acel13281-bib-0053] Lewis, K. N. , Wason, E. , Edrey, Y. H. , Kristan, D. M. , Nevo, E. , & Buffenstein, R. (2015). Regulation of Nrf2 signaling and longevity in naturally long‐lived rodents. Proceedings of the National Academy of Sciences, 112(12), 3722–3727. 10.1073/pnas.1417566112 PMC4378420 25775529

[acel13281-bib-0054] Li, C. , Zhao, R. , Gao, K. , Wei, Z. , Yaoyao Yin, M. , Ting Lau, L. , Chui, D. , & Cheung Hoi Yu, A. (2011). Astrocytes: Implications for neuroinflammatory pathogenesis of Alzheimers disease. Current Alzheimer Research, 8(1), 67–80. 10.2174/156720511794604543 21143158

[acel13281-bib-0055] Li, J. , O, W. , Li, W. , Jiang, Z.‐G. , & Ghanbari, H. (2013). Oxidative stress and neurodegenerative disorders. International Journal of Molecular Sciences, 14(12), 24438–24475. 10.3390/ijms141224438 24351827 PMC3876121

[acel13281-bib-0056] Liddelow, S. A. , Guttenplan, K. A. , Clarke, L. E. , Bennett, F. C. , Bohlen, C. J. , Schirmer, L. , Bennett, M. L. , Münch, A. E. , Chung, W.‐S. , Peterson, T. C. , Wilton, D. K. , Frouin, A. , Napier, B. A. , Panicker, N. , Kumar, M. , Buckwalter, M. S. , Rowitch, D. H. , Dawson, V. L. , Dawson, T. M. , … Barres, B. A. (2017). Neurotoxic reactive astrocytes are induced by activated microglia. Nature, 541(7638), 481–487. 10.1038/nature21029 28099414 PMC5404890

[acel13281-bib-0057] Liu, B. , & Zhou, Z. (2008). Lamin A/C, laminopathies and premature ageing. Histology and Histopathology, 23(6), 747–763. 10.14670/HH-23.747 18366013

[acel13281-bib-0058] Lobsiger, C. S. , & Cleveland, D. W. (2007). Glial cells as intrinsic components of non‐cell‐autonomous neurodegenerative disease. Nature Neuroscience, 10(11), 1355–1360. 10.1038/nn1988 17965655 PMC3110080

[acel13281-bib-0059] Lord, C. L. , Timney, B. L. , Rout, M. P. , & Wente, S. R. (2015). Altering nuclear pore complex function impacts longevity and mitochondrial function in S. cerevisiae. Journal of Cell Biology, 208(6), 729–744. 10.1083/jcb.201412024 PMC4362458 25778920

[acel13281-bib-0060] Lu, T. , Pan, Y. , Kao, S.‐Y. , Li, C. , Kohane, I. , Chan, J. , & Yankner, B. A. (2004). Gene regulation and DNA damage in the ageing human brain. Nature, 429(6994), 883–891. 10.1038/nature02661 15190254

[acel13281-bib-0061] Lukiw, W. J. , Bjattacharjee, S. , Zhao, Y. , Pogue, A. I. , & Percy, M. E. (2012). Generation of reactive oxygen species (ROS) and pro‐inflammatory signaling in human brain cells in primary culture. Journal of Alzheimer’s Disease & Parkinsonism. Suppl 2(001), 10.4172/2161-0460.S2-0011 PMC3947825 24619568

[acel13281-bib-0062] Lynch, A. M. , Murphy, K. J. , Deighan, B. F. , O'Reilly, J. A. , Gun'ko, Y. K. , Cowley, T. R. , Gonzalez‐Reyes, R. E. , & Lynch, M. A. (2010). The impact of glial activation in the aging brain. Aging and Disease, 1, 262–278. 22396865 PMC3295033

[acel13281-bib-0063] Lynch, M. A. (2010). Age‐related neuroinflammatory changes negatively impact on neuronal function. Frontiers in Aging Neuroscience, 1 10.3389/neuro.24.006.2009 PMC2874409 20552057

[acel13281-bib-0064] Mackenzie, I. R. A. , Rademakers, R. , & Neumann, M. (2010). TDP‐43 and FUS in amyotrophic lateral sclerosis and frontotemporal dementia. The Lancet Neurology, 9(10), 995–1007. 10.1016/S1474-4422(10)70195-2 20864052

[acel13281-bib-0065] Maleszewska, M. , Mawer, J. S. P. , & Tessarz, P. (2016). Histone modifications in ageing and lifespan regulation. Current Molecular Biology Reports, 2(1), 26–35. 10.1007/s40610-016-0031-9

[acel13281-bib-0066] Martínez, P. , Ferrara‐Romeo, I., Flores, J. M. , & Blasco, M. A. (2014). Essential role for the TRF2 telomere protein in adult skin homeostasis. Aging Cell, 13(4), 656–668. 10.1111/acel.12221 24725274 PMC4326939

[acel13281-bib-0067] Matias, I. , Morgado, J. , & Gomes, F. C. A. (2019). Astrocyte heterogeneity: Impact to brain aging and disease. Frontiers in Aging Neuroscience, 11, 59 10.3389/fnagi.2019.00059 30941031 PMC6433753

[acel13281-bib-0068] Mertens, J. , Paquola, A. C. M. , Ku, M. , Hatch, E. , Böhnke, L. , Ladjevardi, S. , McGrath, S. , Campbell, B. , Lee, H. , Herdy, J. R. , Gonçalves, J. T. , Toda, T. , Kim, Y. , Winkler, J. , Yao, J. , Hetzer, M. W. , & Gage, F. H. (2015). Directly reprogrammed human neurons retain aging‐associated transcriptomic signatures and reveal age‐related nucleocytoplasmic defects. Cell Stem Cell, 17(6), 705–718. 10.1016/J.STEM.2015.09.001 26456686 PMC5929130

[acel13281-bib-0069] Mertens, J. , Reid, D. , Lau, S. , Kim, Y. , & Gage, F. H. (2018). Aging in a dish: iPSC‐derived and directly induced neurons for studying brain aging and age‐related neurodegenerative diseases. Annual Review of Genetics, 52(1), 271–293. 10.1146/annurev-genet-120417-031534 PMC6415910 30208291

[acel13281-bib-0070] Meyer, K. , Ferraiuolo, L. , Miranda, C. J. , Likhite, S. , McElroy, S. , Renusch, S. , Ditsworth, D. , Lagier‐Tourenne, C. , Smith, R. A. , Ravits, J. , Burghes, A. H. , Shaw, P. J. , Cleveland, D. W. , Kolb, S. J. , & Kaspar, B. K. (2014). Direct conversion of patient fibroblasts demonstrates non‐cell autonomous toxicity of astrocytes to motor neurons in familial and sporadic ALS. Proceedings of the National Academy of Sciences, 111(2), 829–832. 10.1073/pnas.1314085111 PMC3896192 24379375

[acel13281-bib-0071] Miller, J. D. , Ganat, Y. M. , Kishinevsky, S. , Bowman, R. L. , Liu, B. , Tu, E. Y. , Mandal, P. K. , Vera, E. , Shim, J.‐W. , Kriks, S. , Taldone, T. , Fusaki, N. , Tomishima, M. J. , Krainc, D. , Milner, T. A. , Rossi, D. J. , & Studer, L. (2013). Human iPSC‐based modeling of late‐onset disease via progerin‐induced aging. Cell Stem Cell, 13(6), 691–705. 10.1016/J.STEM.2013.11.006 24315443 PMC4153390

[acel13281-bib-0072] Mukherjee, A. B. , & Weinstein, M. E. (1986). Culture media variation as related to in vitro aging of human fibroblasts: I. Effects on population doubling, nuclear volume and nuclear morphology. Mechanisms of Ageing and Development, 37(1), 55–67. 10.1016/0047-6374(86)90118-1 3821189

[acel13281-bib-0073] Niedzielska, E. , Smaga, I. , Gawlik, M. , Moniczewski, A. , Stankowicz, P. , Pera, J. , & Filip, M. (2016). Oxidative stress in neurodegenerative diseases. Molecular Neurobiology, 53(6), 4094–4125. 10.1007/s12035-015-9337-5 26198567 PMC4937091

[acel13281-bib-0074] Ocampo, A. , Reddy, P. , Martinez‐Redondo, P. , Platero‐Luengo, A. , Hatanaka, F. , Hishida, T. , Li, M. , Lam, D. , Kurita, M. , Beyret, E. , & Araoka, T. (2016). In vivo amelioration of age‐associated hallmarks by partial reprogramming. Cell, 167(7), 1719–1733. 10.1016/j.cell.2016.11.052 27984723 PMC5679279

[acel13281-bib-0075] Oksanen, M. , Lehtonen, S. , Jaronen, M. , Goldsteins, G. , Hämäläinen, R. H. , & Koistinaho, J. (2019) Astrocyte alterations in neurodegenerative pathologies and their modeling in human induced pluripotent stem cell platforms. Cellular and Molecular Life Sciences. 76(14), 2739–2760. 10.1007/s00018-019-03111-7 31016348 PMC6588647

[acel13281-bib-0076] Onishi, K. , & Zandstra, P. W. (2015). LIF signaling in stem cells and development. Development, 142(13), 2230–2236. 10.1242/dev.117598 26130754

[acel13281-bib-0077] Ortmann, D. , & Vallier, L. (2017). Variability of human pluripotent stem cell lines. Current Opinion in Genetics & Development, 46, 179–185. 10.1016/j.gde.2017.07.004 28843810

[acel13281-bib-0078] Osorio, F. G. , Soria‐Valles, C. , Santiago‐Fernández, O. , Freije, J. M. P. , & López‐Otín, C. (2016). NF‐κB signaling as a driver of ageing. International Review of Cell and Molecular Biology, 326, 133–174. 10.1016/bs.ircmb.2016.04.003 27572128

[acel13281-bib-0079] Paladino, S. , Conte, A. , Caggiano, R. , Pierantoni, G. M. , & Faraonio, R. (2018). Nrf2 Pathway in age‐related neurological disorders: Insights into MicroRNAs. Cellular Physiology and Biochemistry, 47(5), 1951–1976. 10.1159/000491465 29969760

[acel13281-bib-0080] Parpura, V. , Heneka, M. T. , Montana, V. , Oliet, S. H. R. , Schousboe, A. , Haydon, P. G. , Stout, R. F. , Spray, D. C. , Reichenbach, A. , Pannicke, T. , Pekny, M. , Pekna, M. , Zorec, R. , & Verkhratsky, A. (2012). Glial cells in (patho)physiology. Journal of Neurochemistry, 121(1), 4–27. 10.1111/j.1471-4159.2012.07664.x 22251135 PMC3304021

[acel13281-bib-0081] Patterson, M. , Chan, D. N. , Ha, I. , Case, D. , Cui, Y. , Handel, B. V. , Mikkola, H. K. A. , & Lowry, W. E. (2012). Defining the nature of human pluripotent stem cell progeny. Cell Research, 22(1), 178–193. 10.1038/cr.2011.133 21844894 PMC3351932

[acel13281-bib-0082] Pienta, K. J. , Getzenberg, R. H. , & Coffey, D. S. (1992). Characterization of nuclear morphology and nuclear matrices in ageing human fibroblasts. Mechanisms of Ageing and Development, 62(1), 13–24. 10.1016/0047-6374(92)90140-9 1560681

[acel13281-bib-0083] Pineau, I. , & Lacroix, S. (2007). Proinflammatory cytokine synthesis in the injured mouse spinal cord: Multiphasic expression pattern and identification of the cell types involved. The Journal of Comparative Neurology, 500(2), 267–285. 10.1002/cne.21149 17111361

[acel13281-bib-0084] Re, D. B. , Le Verche, V. , Yu, C. , Amoroso, M. W. , Politi, K. A. , Phani, S. , Ikiz, B. , Hoffmann, L. , Koolen, M. , Nagata, T. , Papadimitriou, D. , Nagy, P. , Mitsumoto, H. , Kariya, S. , Wichterle, H. , Henderson, C. E. , & Przedborski, S. (2014). Necroptosis drives motor neuron death in models of both sporadic and familial ALS. Neuron, 81(5), 1001–1008. 10.1016/j.neuron.2014.01.011 24508385 PMC3951532

[acel13281-bib-0085] Rea, I. M. , Gibson, D. S. , McGilligan, V. , McNerlan, S. E. , Alexander, H. D. , & Ross, O. A. (2018). Age and age‐related diseases: Role of inflammation triggers and cytokines. Frontiers in Immunology, 9 10.3389/fimmu.2018.00586 PMC5900450 29686666

[acel13281-bib-0086] Ribezzo, F. , Shiloh, Y. , & Schumacher, B. (2016). Systemic DNA damage responses in aging and diseases. Seminars in Cancer Biology, 37–38, 26–35. 10.1016/j.semcancer.2015.12.005 PMC4886830 26773346

[acel13281-bib-0087] Rizvi, S. , Raza, S. T. , & Mahdi, F. (2015). Telomere length variations in aging and age‐related diseases. Current Aging Science, 7(3), 161–167. 10.2174/1874609808666150122153151 25612739

[acel13281-bib-0088] Sardo, V. L. , Ferguson, W. , Erikson, G. A. , Topol, E. J. , Baldwin, K. K. , & Torkamani, A. (2017). The effect of aging on human induced pluripotent stem cells. Nature Biotechnology, 35, 69 10.1002/jmri.23741.Proton PMC5505172 27941802

[acel13281-bib-0089] Sarkar, T. J. , Quarta, M. , Mukherjee, S. , Colville, A. , Paine, P. , Doan, L. , Tran, C. M. , Chu, C. R. , Horvath, S. , Qi, L. S. , Bhutani, N. , Rando, T. A. , & Sebastiano, V. (2020). Transient non‐integrative expression of nuclear reprogramming factors promotes multifaceted amelioration of aging in human cells. Nature Communications, 11(1). 10.1038/s41467-020-15174-3 PMC7093390 32210226

[acel13281-bib-0090] Scaffidi, P. , Gordon, L. , & Misteli, T. (2005). The cell nucleus and aging: Tantalizing clues and hopeful promises. PLoS Biology, 3(11), e395 10.1371/journal.pbio.0030395 16277559 PMC1283398

[acel13281-bib-0091] Sheng, C. , Jungverdorben, J. , Wiethoff, H. , Lin, Q. , Flitsch, L. J. , Eckert, D. , Hebisch, M. , Fischer, J. , Kesavan, J. , Weykopf, B. , Schneider, L. , Holtkamp, D. , Beck, H. , Till, A. , Wüllner, U. , Ziller, M. J. , Wagner, W. , Peitz, M. , & Brüstle, O. (2018). A stably self‐renewing adult blood‐derived induced neural stem cell exhibiting patternability and epigenetic rejuvenation. Nature Communications, 9(1). 10.1038/s41467-018-06398-5 PMC6168501 30279449

[acel13281-bib-0092] Shibata, N. , & Kobayashi, M. (2008). The role for oxidative stress in neurodegenerative diseases. Brain and Nerve, 60, 157–170. 10.5607/en.2015.24.4.325 18306664

[acel13281-bib-0093] Sigfridsson, E. , Marangoni, M. , Johnson, J. A. , Hardingham, G. E. , Fowler, J. H. , & Horsburgh, K. (2018) Astrocyte‐specific overexpression of Nrf2 protects against optic tract damage and behavioural alterations in a mouse model of cerebral hypoperfusion. Scientific Reports. 8(1). 10.1038/s41598-018-30675-4 PMC6105641 30135571

[acel13281-bib-0094] Simpson, J. E. , Ince, P. G. , Shaw, P. J. , Heath, P. R. , Raman, R. , Garwood, C. J. , Gelsthorpe, C. , Baxter, L. , Forster, G. , Matthews, F. E. , Brayne, C. , & Wharton, S. B. (2011). Microarray analysis of the astrocyte transcriptome in the aging brain: Relationship to Alzheimer’s pathology and APOE genotype. Neurobiology of Aging, 32(10), 1795–1807. 10.1016/j.neurobiolaging.2011.04.013 21705112

[acel13281-bib-0095] Smogorzewska, A. , van Steensel, B. , Bianchi, A. , Oelmann, S. , Schaefer, M. R. , Schnapp, G. , & de Lange, T. (2000). Control of human telomere length by TRF1 and TRF2. Molecular and Cellular Biology, 20(5), 1659–1668. 10.1128/mcb.20.5.1659-1668.2000 10669743 PMC85349

[acel13281-bib-0096] Sofroniew, M. V. , & Vinters, H. V. (2010). Astrocytes: biology and pathology. Acta Neuropathologica, 119(1), 7–35. 10.1007/s00401-009-0619-8 20012068 PMC2799634

[acel13281-bib-0097] Soreq, L. , Rose, J. , Soreq, E. , Hardy, J. , Trabzuni, D. , Cookson, M. R. , Smith, C. , Ryten, M. , Patani, R. , & Ule, J. (2017). Major shifts in glial regional identity are a transcriptional hallmark of human brain aging. Cell Reports, 18(2), 557–570. 10.1016/j.celrep.2016.12.011 28076797 PMC5263238

[acel13281-bib-0098] Stade, K. , Ford, C. S. , Guthrie, C. , & Weis, K. (1997). Exportin 1 (Crm1p) is an essential nuclear export factor. Cell, 90(6), 1041–1050. 10.1016/S0092-8674(00)80370-0 9323132

[acel13281-bib-0099] Stegeman, R. , & Weake, V. M. (2017). Transcriptional signatures of aging. Journal of Molecular Biology, 429(16), 2427–2437. 10.1016/j.jmb.2017.06.019 28684248 PMC5662117

[acel13281-bib-0100] Takahashi, K. , Tanabe, K. , Ohnuki, M. , Narita, M. , Ichisaka, T. , Tomoda, K. , & Yamanaka, S. (2007). Induction of pluripotent stem cells from adult human fibroblasts by defined factors. Cell, 131(5), 861–872. 10.1016/J.CELL.2007.11.019 18035408

[acel13281-bib-0101] Tang, Y. U. , Liu, M.‐L. , Zang, T. , & Zhang, C.‐L. (2017). Direct reprogramming rather than iPSC‐based reprogramming maintains aging hallmarks in human motor neurons. Frontiers in Molecular Neuroscience. Frontiers, 10, 359 10.3389/fnmol.2017.00359 PMC5676779 29163034

[acel13281-bib-0102] Tchieu, J. , Calder, E. L. , Guttikonda, S. R. , Gutzwiller, E. M. , Aromolaran, K. A. , Steinbeck, J. A. , Goldstein, P. A. , & Studer, L. (2019). NFIA is a gliogenic switch enabling rapid derivation of functional human astrocytes from pluripotent stem cells. Nature Biotechnology, 37(3), 267–275. 10.1038/s41587-019-0035-0 PMC6591152 30804533

[acel13281-bib-0103] Tilstra, J. S. , Clauson, C. L. , Niedernhofer, L. J. , & Robbins, P. D. (2011). NF‐κB in aging and disease. Aging and Disease, 2, 449–465. 22396894 PMC3295063

[acel13281-bib-0104] van Horssen, J. , Schreibelt, G. , Drexhage, J. , Hazes, T. , Dijkstra, C. D. , van der Valk, P. , & de Vries, H. E. (2008). Severe oxidative damage in multiple sclerosis lesions coincides with enhanced antioxidant enzyme expression. Free Radical Biology and Medicine, 45(12), 1729–1737. 10.1016/J.FREERADBIOMED.2008.09.023 18930811

[acel13281-bib-0105] Varcianna, A. , Myszczynska, M. A. , Castelli, L. M. , O'Neill, B. , Kim, Y. , Talbot, J. , Nyberg, S. , Nyamali, I. , Heath, P. R. , Stopford, M. J. , Hautbergue, G. M. , & Ferraiuolo, L. (2019). Micro‐RNAs secreted through astrocyte‐derived extracellular vesicles cause neuronal network degeneration in C9orf72 ALS. EBioMedicine, 40, 626–635. 10.1016/j.ebiom.2018.11.067 30711519 PMC6413467

[acel13281-bib-0106] Verkhratsky, A. , & Nedergaard, M. (2016). The homeostatic astroglia emerges from evolutionary specialization of neural cells. Philosophical Transactions of the Royal Society B: Biological Sciences, 371(1700), 20150428 10.1098/rstb.2015.0428 PMC4938028 27377722

[acel13281-bib-0107] Victor, M. B. , Richner, M. , Olsen, H. E. , Lee, S. W. , Monteys, A. M. , Ma, C. , Huh, C. J. , Zhang, B. O. , Davidson, B. L. , Yang, X. W. , & Yoo, A. S. (2018). Striatal neurons directly converted from Huntington’s disease patient fibroblasts recapitulate age‐associated disease phenotypes. Nature Neuroscience, 21(3), 341–352. 10.1038/s41593-018-0075-7 29403030 PMC5857213

[acel13281-bib-0108] Waller, R. , Woodroofe, M. N. , Francese, S. , Heath, P. R. , Wharton, S. B. , Ince, P. G. , Sharrack, B. , & Simpson, J. E. (2012). Isolation of enriched glial populations from post‐mortem human CNS material by immuno‐laser capture microdissection. Journal of Neuroscience Methods, 208(2), 108–113. 10.1016/j.jneumeth.2012.04.014 22609336

[acel13281-bib-0109] Webster, C. P. , Smith, E. F. , Bauer, C. S. , Moller, A. , Hautbergue, G. M. , Ferraiuolo, L. , Myszczynska, M. A. , Higginbottom, A. , Walsh, M. J. , Whitworth, A. J. , & Kaspar, B. K. (2016). The C9orf72 protein interacts with Rab1a and the ULK1 complex to regulate initiation of autophagy. The EMBO Journal, 35(15), 1656–1676. 10.15252/embj.201694401 27334615 PMC4969571

[acel13281-bib-0110] Wu, Y. , Zhang, A.‐Q. , & Yew, D. T. (2005). Age related changes of various markers of astrocytes in senescence‐accelerated mice hippocampus. Neurochemistry International, 46(7), 565–574. 10.1016/J.NEUINT.2005.01.002 15843051

[acel13281-bib-0111] Zhong, Y. , Wang, J. , Henderson, M. J. , Yang, P. , Hagen, B. M. , Siddique, T. , Vogel, B. E. , Deng, H.‐X. , & Fang, S. (2017). Nuclear export of misfolded SOD1 mediated by a normally buried NES‐like sequence reduces proteotoxicity in the nucleus. eLife, 6 10.7554/eLife.23759 PMC5449186 28463106

