# Supplementary material for: Directly converted astrocytes retain the ageing features of the donor fibroblasts and elucidate the astrocytic contribution to human CNS health and disease
Source: Aging Cell. 2020 Dec 13;20(1):e13281. doi: 10.1111/acel.13281 (PMC7811849; doi:10.1111/acel.13281)
Supplement: Supplementary file 1 — Supinfo [file ACEL-20-e13281-s001.docx]

**Supplementary Information**

**Cell Culture**

Human skin fibroblast samples were obtained from different sources (Table S1). Prof. Pamela Shaw and Dr Daniel Blackburn provided the fibroblast samples from the University of Sheffield (Study number STH16573, Research Committee reference 12/YH/0330 and MODEL-AD research study number STH19080 Research and Ethics Committee number: 16/YH/0155 respectively). Human skin fibroblast samples from donors below the age of consent were from established tissue banks (Coriell Institute) as shown in Table S1. Informed consent was obtained from all subjects before sample collection.

100,000 fibroblasts were seeded in a well of a six-well plate and treated with retroviral vectors for *OCT3, SOX2*, *KLF4* and *c-MYC* (Meyer *et al.*, 2014). After 48h the medium was switched to NPC conversion medium, consisting of DMEM/F12, 1% N2, 1% B27, EGF (40ng/ml) and FGF (20 ng/mL). Typically, after 2-4 days 60-80% of the fibroblasts changed shape to smaller morphology; some lifted and created sphere-like structures, some started proliferating in rosettes or simply remained attached and changed morphology. These cells proliferated quickly and were split when 100% confluent. These were collected or lifted using Accutase (StemPro® Accutase® Cell Dissociation Reagent, Gibco) and, depending on the density and their growth rate, they were expanded in multiple wells of a six-well plate or a 10cm dish coated with human fibronectin (5 μg/mL; Millipore) over a period of 18-21 days. After this first expansion period, during which the fibroblasts that did not reprogram were diluted and eventually lost, cells were stained for Nestin and PAX6 to establish successful iNPC conversion. Typically, successful conversion is defined as yielding >95% cell positive for PAX6 and Nestin. This pure iNPC population was then expanded and stored for up to 18 passages. The lines used in this study all yielded a purity >98% regardless of the age of the donor (Figure S1).

The human induced pluripotent stem cells (iPSC) line used in this study named control MIFF1 (Desmarais JA et al., 2016) was kindly provided by Professor Peter Andrews and Dr. Ivana Barbaric (Centre for Stem Cell Biology, The University of Sheffield). iPSCs were maintained in complete mTeSR™-Plus™ Medium (StemCell Technologies) in Matrigel® growth factor reduced-coated plates (Corning®) according to the manufacturer’s recommendations. Cultures were replenished with fresh medium every alternate day. The passage of cells was performed when the cells reached around 70% of confluence, every four to six days as clumps using ReLeSR™ an enzyme-free reagent for dissociation (StemCell Technologies) according to the manufacturer’s recommendations. For all the experiments in this study, iPSCs were used between passage 20 and 28, all iPSCs were cultured in 5% O_2_, 5% CO_2_ at 37°C.

To induce differentiation, iPSCs were transferred for matrigel-coated plate (Corning® Matrigel® Growth Factor Reduced). On the day after plating (day 1), after the cells had reached ∼100% confluence, the cells were washed once with PBS and then the medium was replaced with neural medium (50% of KnockOut™ DMEM/F-12, 50 % of Neurobasal (ThermoFisher), 0.5× N2 supplement (ThermoFisher), 1x Gibco® GlutaMAX™ Supplement (ThermoFisher), 0.5x B-27 (ThermoFisher), 50 U ml^−1^ penicillin and 50 mg ml^−1^ streptomycin, supplemented with DMH-1 2 μM (Tocris), SB431542-10 μM (Tocris) and CHIR99021 3 μM (CHIR, Tocris). The medium was changed every day for 6 days. On day 7, the medium was replaced with neural medium supplemented with SB431542-10 μM, and CHIR 1 μM, DMH-1 2 μM, all-trans retinoic acid 0.1 μM (RA, StemCell Technologies), and purmorphamine 0.5 μM (PMN, Tocris). The medium was changed every day until day 12 when was possible to see a uniform neuroepithelial sheet. On day 12 the cells were split 1:6 with accutase (StemPro® Accutase® Cell Dissociation Reagent, Gibco), onto a matrigel-coated plate in the presence of 10 µM of rock inhibitor (Y-27632 dihydrochloride, Tocris), giving rise to a sheet of NPC.  After 24 hours of incubation, the medium was changed for neural medium supplemented with RA 0.5 μM and PMN 0.1 μM. The medium was changed every day until day 19 when the motor neuron progenitors (MNP) can be visualized. On day 20 MNP were split with accutase onto to matrigel-coated plates and the medium was replaced with neural medium supplemented with compound E 0.1 µM (Cpd E, Tocris), retinoic acid 0.5 μM, PMN 0.1 μM, BDNF 10ng/mL (ThermoFisher), CNTF 10ng/mL (ThermoFisher) and IGF 10ng/mL (ThermoFisher). At this stage the cells were fed every other day with neuronal medium until day 40. This protocol typically yields ~90% ChAT^+^ MNs.

***Table S1.*** *Summary of the information on the astrocytes (iNPC-As) and fibroblast lines used in this study*

| **ID** | **Cell line** | **Age** | **Gender** | **Biobank** |
| --- | --- | --- | --- | --- |
| Young 1(Y1) | GM08680 | 5 months | Male | Coriell |
| Young 2 (Y2) | GM00498 | 3 years | Male | Coriell |
| Young 3 (Y3) | GM03813 | 3 years | Male | Coriell |
| Old 1 (O1) | MAD14 | 56 years | Male | Blackburn |
| Old 2 (O2) | 3050 | 55 years | Male | Shaw |
| Old 3 (O3) | 155 | 42 years | Male | Shaw |

**
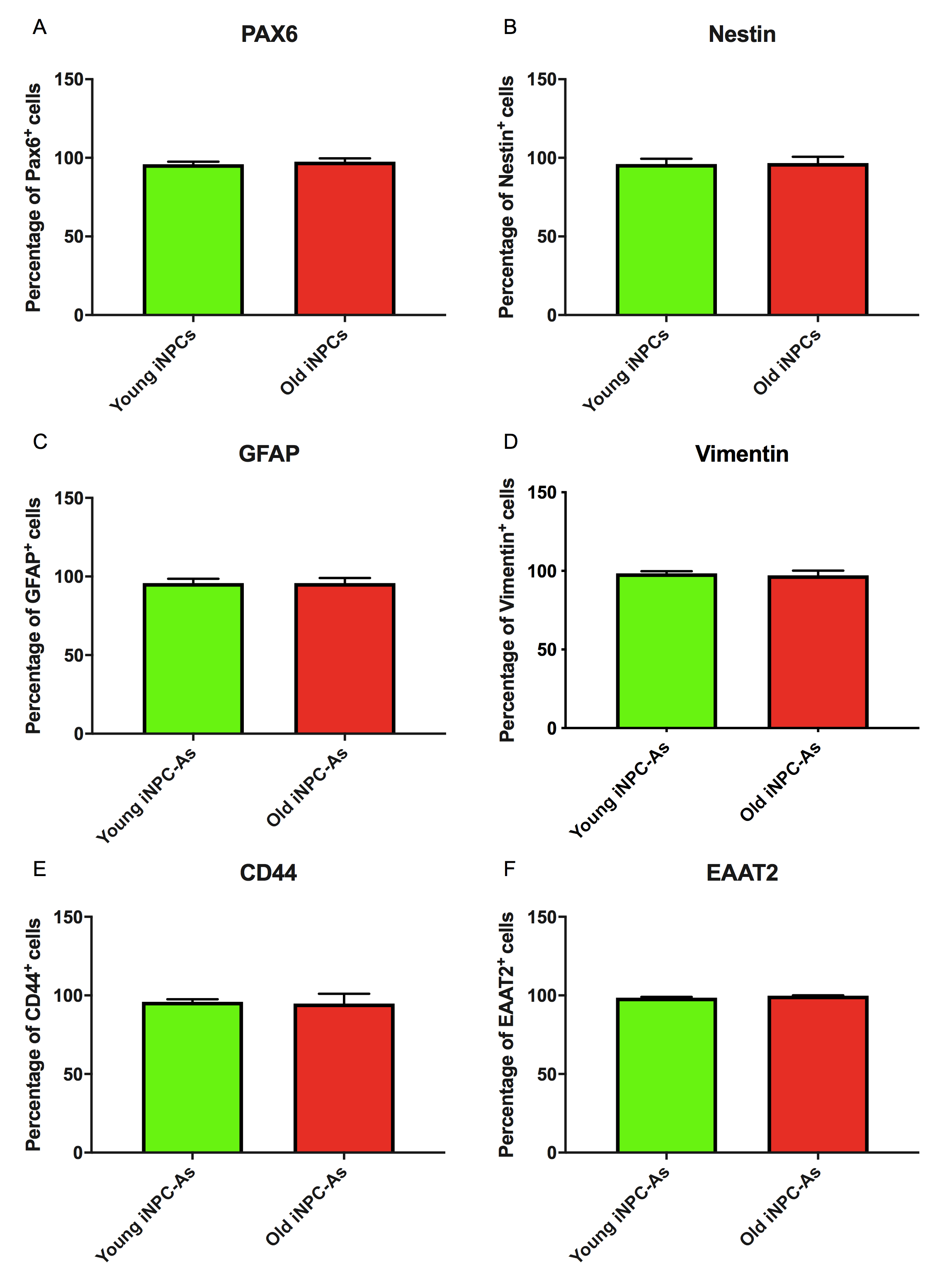
Figure S1 related to Figure 1 Characterization of iNPC-As from old versus young donors**

(A) (B) Percentage of cells expressing iNPC markers PAX6 and Nestin upon fibroblast reprogramming

(C) (D) (E) (F) Percentage of cells expressing astrocytic markers (GFAP, Vimentin (VIM), CD44 and EAAT2) in young and old donor derived iNPC-As (n=3) after differentiation from iNPCs.

***Table S2.*** *Summary of the information on microarray data used in this study*

| **ID** | **Origin** | **Age** | **Gender** | **GEO accession number** |
| --- | --- | --- | --- | --- |
| *Fetal_1* | Sciencell | - | M/F | 10.15131/shef.data.12162282 |
| *Fetal_2* | Sciencell | - | M/F | 10.15131/shef.data.12162282 |
| *Fetal_3* | Sciencell | - | M/F | 10.15131/shef.data.12162282 |
| *PM_1* | Laser-captured from brain | 72 years | F | GSE83670 |
| *PM_2* | Laser-captured from brain | 75 years | M | GSE83670 |
| *PM_3* | Laser-captured from brain | 78 years | F | GSE83670 |
| *PM_4* | Laser-captured from brain | 78 years | F | GSE83670 |
| *Old iNPC-As_1* | Direct reprogramming from skin fibroblasts | 55 years | F | GSE87385 |
| *Old iNPC-As_2* | Direct reprogramming from skin fibroblasts | 69 years | F | GSE87385 |
| *Old iNPC-As _3* | Direct reprogramming from skin fibroblasts | 47 years | M | GSE87385 |
| *Young iNPC-As _1* | Direct reprogramming from skin fibroblasts | 3 years | F | 10.15131/shef.data.12162282 |
| *Young*  *iNPC-As _2* | Direct reprogramming from skin fibroblasts | 8 years | F | 10.15131/shef.data.12162282 |
| *Young*  *iNPC-As_3* | Direct reprogramming from skin fibroblasts | 5 months | M | 10.15131/shef.data.12162282 |

**
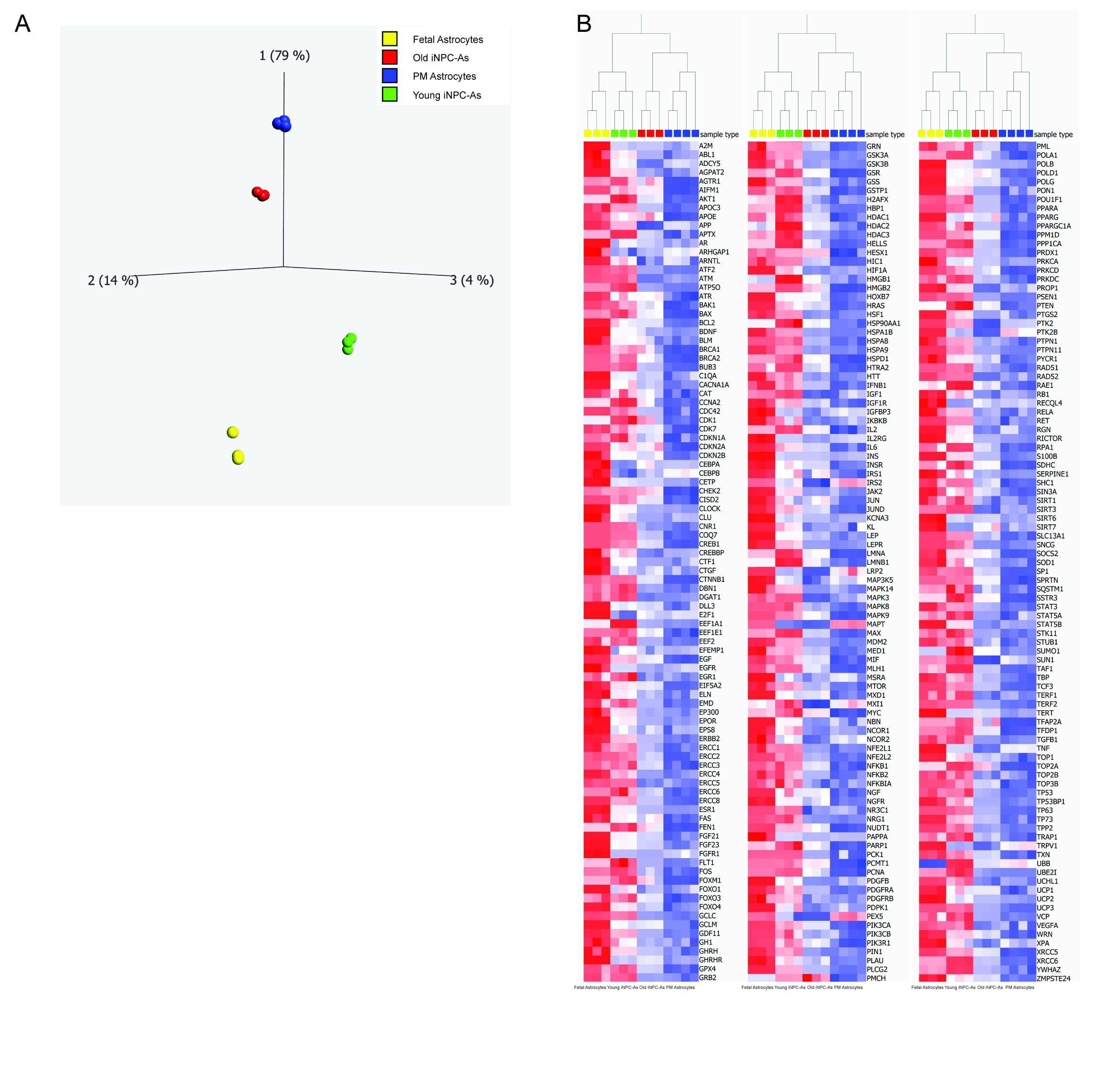
Figure S2 related to Figure 2 Old donor iNPC-As and post-mortem (PM) astrocytes have distinctly different transcriptomes to fetal astrocytes and young donor iNPC-As**

(A) Principal component analysis (PCA) plot of fetal astrocytes, PM astrocytes and old and young donor derived iNPC-As. Multi-group comparison, p≤1x10e-4.

(B) Hierarchical cluster heat-map of fetal astrocytes, PM astrocytes and old and young donor iNPC-As. Multi-group comparison, p≤1x10e-4.

***Table S3*** *Related to Figure 2* *Genes in the intersection between the two comparisons (excel file table S3)*

**
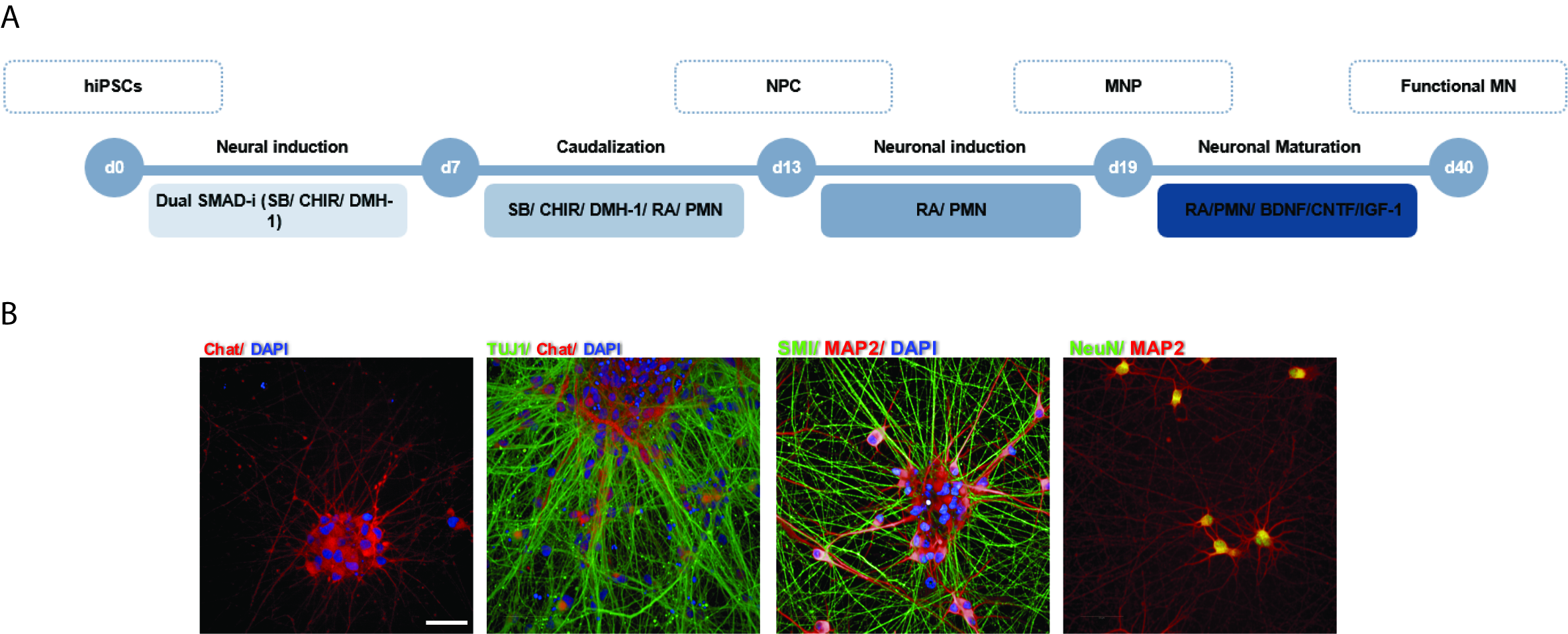
**

**Figure S3 MN differentiation**

(A) Schematic illustration of the differentiation protocol for human MN derived from hiPSCs.

(B) Representative images of characterisation of MN with cell-specific markers Tuj1, Chat, SMI32, MAP2 and NeuN.


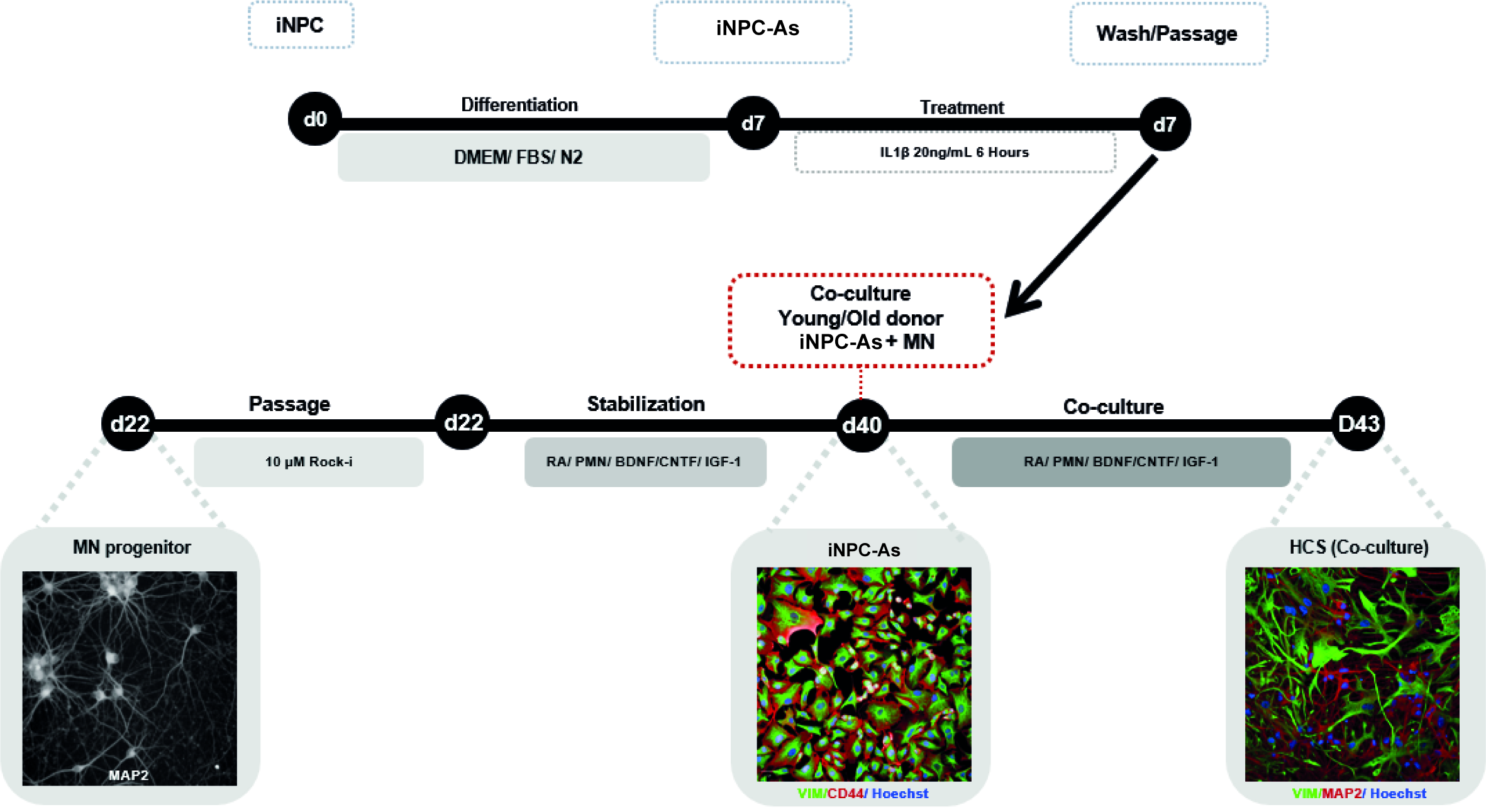


**Figure S4 Human co-culture system workflow.**

(A) Schematic illustration of the co-culture representing the differentiation of the iNPC-As from young and old donors. After the differentiation, astrocytes were exposed to human IL-1β (20ng/mL) for 6 hours. After this treatment, the cells were washed and then seeded on to MNs. MN progenitors were seeded and on day 40 astrocytes were added on the MNs. The co-culture was kept for 72 hours.

***
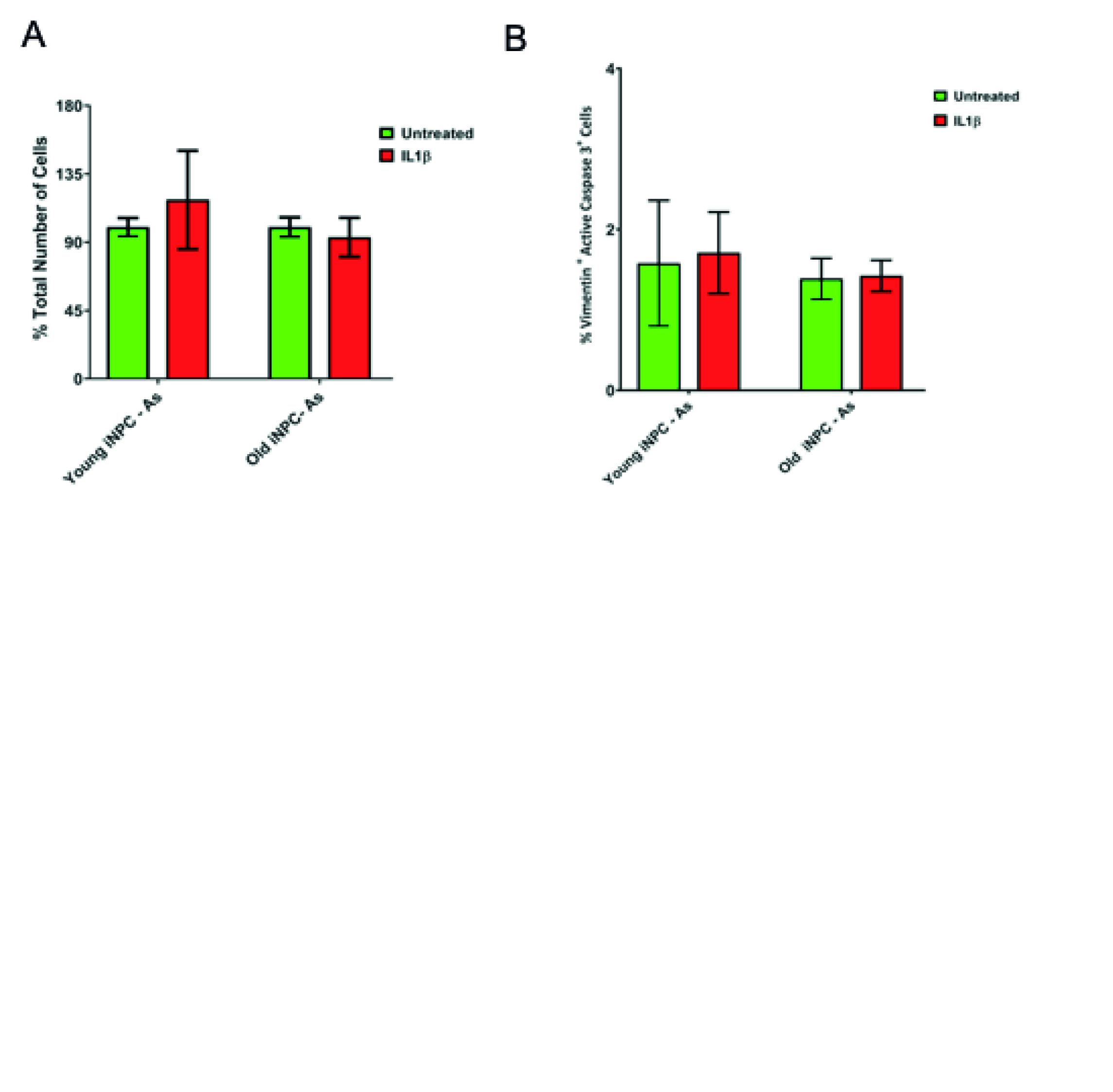
*Figure S5 Effect of IL-1β on iNPC-As survival.**

(A) Graph representing number of nuclei normalised to untreated condition (untreated condition of each age group = 100%) in young and old iNPC-As with and without IL1β treatment for 6h in monoculture and counted 72h later to mirror co-culture conditions.

(B) Percentage of Vim+/Casp3+ iNPC-As normalised to the total number of Vim+ cells in co-culture. Two-way ANOVA, ****P<0.0001 (n=3).

***Table S4****. Primer sequences used for q-PCR, Forward (Fw) and Reverse (Rv)*

| **Primer** | **Sequence (5’-3’)** |
| --- | --- |
| B-Actin_Fw | TCCCCCAACTTGAGATGTATGAAG |
| B-Actin_Rv | AACTGGTCTCAAGTCAGTGTACAGG |
| RLP13A_Fw | CAAGCGGATGAACACCAACC |
| RLP13A_Rv | TTTTGTGGGGCAGCATACCT |
| U1_Fw | CCATGATCACGAAGGTGGTT |
| U1_Rv | ATGCAGTCGAGTTTCCCACA |
| GAPDH_Fw | CAACTTTGGTATCGTGGAAGGAC |
| GAPDH_Rv | ACAGTCTTCTGGATGGCAGTG |
| TERF2_Fw | TTATTCGAGAAAAGAACTTGGCCC |
| TERF2_Rv | TGAGGAGGTAGGGCTCGG |
| RANBP17_Fw | CACTTCGATGCAGAGAGGCTA |
| RANBP17_Rv | CACTGGTTCCGACAGTCTTC |
| LAMA3_Fw | TGTTTAAACTGCAGCCTCCCA |
| LAMA3_Rv | ACACATTTCAAGTTCCCGGC |

***Table S5.*** *Summary of the information on the induced pluripotent stem cells (iPSC) lines used in this study*

| **ID: iPSC Lines** | **Cell line** | **Age** | **Gender** | **Identifier** | **Biobank** |
| --- | --- | --- | --- | --- | --- |
| Young 1(Y1) | MIFF1 | < 1 Month | Male | RRID:CVCL_1E69 | TUoS |
| Young 2 (Y2) | CS00iCTR-nxx | 6 years | Male | RRID:CVCL_UK08 | Cedars-sinai |
| Old 1 (O1) | GM23338 | 55 years | Male | RRID:CVCL_F182 | Coriell |
| Old 2 (O2) | CS14iCTR-nxx | 30-35 years | Female | RRID:CVCL_JK54 | Cedars-sinai |

***Table S6*** *Primary antibodies used in this study*

| **Primary Antibody** | **Source** | **Species** | **Assay (WB or ICC)** | **Dilution used** |
| --- | --- | --- | --- | --- |
| **GAPDH** | Cell Signalling | Rabbit | WB | 1:2000 |
| **NRF2** | Abcam | Rabbit | WB | 1:1000 |
| **SOD1** | Cell Signalling | Rabbit | WB | 1:1000 |
| **XPO1** | Abcam | Rabbit | WB | 1:2000 |
| **RANBP17** | Abcam | Rabbit | WB | 1:2000 |
| **VIMENTIN** | Millipore | Chicken | ICC | 1:1000 |
| **GFAP** | Dako | Rabbit | ICC | 1:1000 |
| **CD44** | Abcam | Rabbit | ICC | 1:200 |
| **EAAT2** | SantaCruz | Goat | ICC | 1:100 |
| **Histone H3**  **(tri methyl K9)** | Abcam | Rabbit | ICC | 1:800 |
| **Lamin A/C** | Abcam | Mouse | ICC | 1:800 |
| **Nestin** | Abcam | Mouse | ICC | 1:200 |
| **Nestin** | Biolegend | Rabbit | ICC | 1:500 |
| **PAX6** | Abcam | Rabbit | ICC | 1:1000 |
| **PAX6** | Millipore | Mouse | ICC | 1:200 |
| **Beta III Tubulin** | Millipore | Chicken | ICC | *1:1000* |
| **Beta III Tubulin** | Biolegend | Mouse | ICC | *1:1000* |
| **NeuN** | Biolegend | Mouse | ICC | *1:1000* |
| **CHAT** | Millipore | Goat | ICC | *1:100* |
| **SMI32** | Biolegend | Mouse | ICC | 1:500 |
| **MAP2** | *Synaptic systems* | *Guinea pig* | ICC | 1:1000 |
| **Caspase 3** | *Millipore* | *Rabbit* | ICC | *1:200* |
| **γH2AX clone JBW301** | *Millipore* | *Mouse* | ICC | *1:500* |

***Table S7****. Secondary antibodies used in this study*

| **Secondary antibody** | **Source** | **Assay** | **Dilution used** |
| --- | --- | --- | --- |
| **Alexa Fluor 488 donkey α-mouse IgG(H+L)** | Invitrogen,ThermoFisher | ICC | 1:400 |
| **Alexa Fluor 488 donkey α-rabbit IgG(H+L)** | Invitrogen,ThermoFisher | ICC | 1:400 |
| **Alexa Fluor 488 goat α-chicken IgG(H+L)** | Invitrogen,ThermoFisher | ICC | 1:1000 |
| **Alexa Fluor 488 goat α-rabbit IgG(H+L)** | Invitrogen,ThermoFisher | ICC | 1:1000 |
| **Alexa Fluor 555 donkey α-goat IgG(H+L)** | Invitrogen,ThermoFisher | ICC | 1:400/1000 |
| **Alexa Fluor 568 donkey α-mouse IgG(H+L)** | Invitrogen,ThermoFisher | ICC | 1:400/1000 |
| **Alexa Fluor 568 donkey α-rabbit IgG(H+L)** | Invitrogen,ThermoFisher | ICC | 1:400 |
| **Alexa Fluor 586 goat α-rabbit IgG(H+L)** | Invitrogen,ThermoFisher | ICC | 1:1000 |
| **Alexa Fluor 594 donkey α-mouse IgG(H+L)** | Invitrogen,ThermoFisher | ICC | 1:400 |
| **Alexa Fluor 647 goat α-guinea pig IgG(H+L)** | Invitrogen,ThermoFisher | ICC | 1:400 |
| **α-Rabbit IgG (H+L), HRP Conjugate** | Promega | WB | 1:5000 |
| **α-mouse IgG (H+L), HRP Conjugate** | Promega | WB | 1:5000 |
| **α-chicken IgGY (H+L), HRP Conjugate** | Invitrogen,ThermoFisher | WB | 1:5000 |
